# Discovery of the cryptic function of terpene cyclases as aromatic prenyltransferases

**DOI:** 10.1038/s41467-020-17642-2

**Published:** 2020-08-07

**Authors:** Haibing He, Guangkai Bian, Corey J. Herbst-Gervasoni, Takahiro Mori, Stephen A. Shinsky, Anwei Hou, Xin Mu, Minjian Huang, Shu Cheng, Zixin Deng, David W. Christianson, Ikuro Abe, Tiangang Liu

**Affiliations:** 1 Graduate School of Pharmaceutical Sciences, University of Tokyo, Tokyo, Japan; 2 Key Laboratory of Combinatorial Biosynthesis and Drug Discovery, Ministry of Education and School of Pharmaceutical Sciences, Wuhan University, Wuhan, China; 3 Roy and Diana Vagelos Laboratories, Department of Chemistry, University of Pennsylvania, Philadelphia, PA 19104-6323 USA; 4 Hubei Engineering Laboratory for Synthetic Microbiology, Wuhan Institute of Biotechnology, Wuhan, China

**Keywords:** Enzyme mechanisms, Enzymes, Proteins, X-ray crystallography

## Abstract

Catalytic versatility is an inherent property of many enzymes. In nature, terpene cyclases comprise the foundation of molecular biodiversity as they generate diverse hydrocarbon scaffolds found in thousands of terpenoid natural products. Here, we report that the catalytic activity of the terpene cyclases AaTPS and FgGS can be switched from cyclase to aromatic prenyltransferase at basic pH to generate prenylindoles. The crystal structures of AaTPS and FgGS provide insights into the catalytic mechanism of this cryptic function. Moreover, aromatic prenyltransferase activity discovered in other terpene cyclases indicates that this cryptic function is broadly conserved among the greater family of terpene cyclases. We suggest that this cryptic function is chemoprotective for the cell by regulating isoprenoid diphosphate concentrations so that they are maintained below toxic thresholds.

## Abstract (web summary)

Terpene cyclases catalyze the formation of diverse hydrocarbon scaffolds found in terpenoids. Here, the authors report the cryptic function of class I terpene cyclases as aromatic prenyltransferases and the universality of this cryptic feature is confirmed using enzymes from different sources.

## Introduction

With more than 80,000 different compounds identified to date, terpenoids represent the largest and most widely distributed family of natural products found on the earth. Terpenoids are extensively used in modern applications such as perfumes and spices, antitumor and antimalaria agents, and as jet fuel^1,2^. Terpenoid biosynthesis begins with the condensation of isopentenyl diphosphate (IPP) and dimethylallyl diphosphate (DMAPP) to form geranyl diphosphate (GPP). The subsequent addition of one or more IPP units yields progressively longer isoprenoid diphosphates such as farnesyl diphosphate (FPP), geranylgeranyl diphosphate (GGPP), and geranylfarnesyl diphosphate (GFPP). Following elongation, the linear isoprenoid diphosphates undergo reactions catalyzed by terpene cyclases to generate diverse terpene hydrocarbon scaffolds with multiple fused rings and stereocenters. Class I terpene cyclases require 3 Mg^2+^ ions for catalytic function, and these metal ions are coordinated by conserved metal-binding motifs in a unique α-helical protein fold^3^. The reactions catalyzed by terpene cyclases are largely responsible for the expansive chemodiversity of terpenoid natural products.

In addition to their utility as substrates for terpene cyclases, isoprenoid diphosphates also serve as co-substrates for aromatic prenyltransferases. These enzymes catalyze the Friedel–Crafts alkylation of aromatic and heteroaromatic rings at different positions to form various prenylated products^4–6^. Aromatic prenyltransferases are found in both membrane-embedded and soluble forms. The structure of the membrane-embedded aromatic prenyltransferase UbiA reveals an α-helical protein fold topologically identical to that of a class I terpene cyclase^4,7^. In contrast, soluble aromatic prenyltransferases adopt a completely distinct protein fold consisting of five repeating αββα motifs; these enzymes are accordingly designated ABBA prenyltransferases^8,9^. Until now, there has been no evidence suggesting that aromatic prenyltransferases or class I terpene cyclases exhibit bifunctional cross-reactivity, such that a class I terpene cyclase could also catalyze an aromatic prenyltransferase reaction or vice versa.

Recently, we established robust platforms in *Escherichia coli* and *Saccharomyces cerevisiae* to accelerate the discovery of C_20_ diterpene and C_25_ sesterterpene natural products with diverse molecular structure and stereochemistry^10,11^. These studies led to the discovery and characterization of the terpene cyclases *Fusarium graminearum* mangicdiene synthase (FgMS), *F. graminearum* GJ1012 synthase (FgGS), *F. graminearum* fusariumdiene and fusagramineol synthase (FgFS), and *Colletotrichum gloeosporioides* dolasta-1(15),8-diene synthase (CgDS); the products generated by each of these enzymes were systematically characterized^11–14^. Since our report of these class I terpene cyclases, we further probed their functional properties and we encountered an unexpected surprise—we discovered substantial cryptic aromatic prenyltransferase activity.

**Fig. 1:**
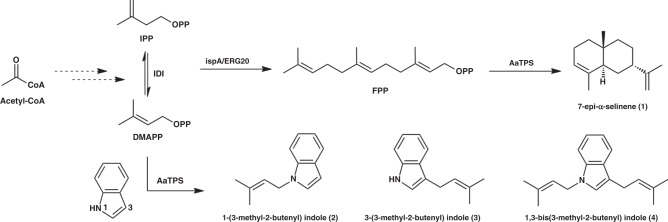
Reaction products generated by AaTPS. Sesquiterpene and prenylindole products generated by AaTPS using FPP as substrate, or indole and DMAPP as co-substrates. IPP isopentenyl diphosphate, DMAPP dimethylallyl diphosphate, FPP farnesyl diphosphate, IDI isopentenyl diphosphate isomerase, ispA/ERG20 FPP synthase.

Here, we describe the discovery that class I terpene cyclases are bifunctional, in that they catalyze both terpene cyclization and aromatic prenyltransferase reactions in the same active site (Fig. 1). Notably, class I terpene cyclases are not evolutionarily related to ABBA-type prenyltransferases, nor are they related to soluble aromatic prenyltransferases from fungi (Supplementary Fig. 1). To provide insight on catalytic mechanism in these bifunctional enzymes, we additionally report crystal structures of the class I terpene cyclases AaTPS and FgGS. These structural and functional studies illuminate a dynamic regulation strategy for the utilization of indole prenylation to mitigate elevated DMAPP concentrations that could otherwise be toxic to the cell.

## Results

### Robust chassis facilitates characterization of AaTPS

**Fig. 2:**
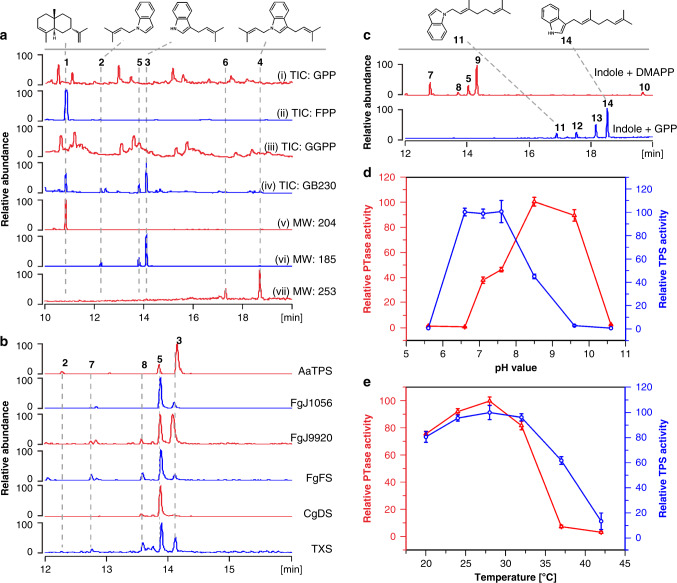
Indole prenyltransferase activities of terpene cyclases. **a** GC/MS chromatogram of terpenes produced by in vitro assay of AaTPS with (i) GPP, (ii) FPP and (iii) GGPP, respectively. Peaks shown in (i) and (iii) correspond to background, (iv), compounds produced by AaTPS overexpression in *E. coli* GB230, (v) sesquiterpene compound **1** produced by *E. coli* GB230, (vi) prenylindoles **2**, **3**, and **5** with molecular weight (MW) 185 produced by *E. coli* GB230, and vii) prenylindoles **4** and **6** with MW 253 produced by *E. coli* GB230. **b**, GC/MS chromatogram of prenylindoles **2**, **3**, **5**, **7**, and **8** with MW 185 produced by AaTPS, FgJ1056, FgJ9920, FgFS, CgDS, and TXS while using indole and DMAPP as co-substrates. **c** GC/MS chromatogram of prenylindoles produced by in vitro assay of FgGS. Prenylindoles **5**, **7**, **8**, **9** with MW 185 and **10** with MW 253 were detected when indole and DMAPP were used as co-substrates, and prenylindoles **11**–**14** with MW 253 were produced when indole and GPP were used as co-substrates. **d** pH-Dependent assays of terpene cyclase and indole prenyltransferase activities of AaTPS. **e** Temperature-dependent assays of terpene synthase and indole prenyltransferase activities of AaTPS. PTase represents prenyltransferase, TPS represents terpene synthase. Relative abundance and relative activities are shown in this figure; the compound with the highest abundance or the enzyme with the highest activity is set as 100. Data represent the means ± standard deviation (SD) of three biological replicates. Source data are provided as a Source Data file.

During the genome mining of terpene synthases, the silent gene *AaTPS* from the filamentous fungi *Alternaria alternata* TPF6 was predicted to be a class I sesquiterpene synthase and was selected for functional characterization. Sequence alignment of AaTPS with other terpene synthases highlighted the highly conserved aspartate-rich motif, D^176^DSWE^180^, and the NSE triad, N^307^DLASYDKE^315^ (Supplementary Fig. 2)^3,15,16^, both of which are required for coordination to the 3 Mg^2+^ ions necessary for catalysis. To confirm the predicted coding sequence of AaTPS, genomic DNA of this gene was put under the control of the *alcA* promoter and transformed into *A. alternata* TPF6 via the *Agrobacterium tumefaciens*-mediated transformation method to generate mutant *Alternaria alternata* GB171^17^. Transcription data were gathered and the mRNA sequence of AaTPS was confirmed. Subsequently, AaTPS (pGB163) was purified and an in vitro assay was performed to evaluate the ability of the enzyme to utilize all-trans-polyisoprenoid diphosphates GPP, FPP, and GGPP as substrates. The in vitro reaction showed that this enzyme can utilize FPP as the sole substrate to produce sesquiterpene compound **1** (Fig. 2a). To obtain this compound, AaTPS was cloned and the resulting plasmid pGB230 was transplanted into the previously reported sesquiterpene overproduction chassis *E. coli* C1^10^ to generate *E. coli* strain GB230, which overproduces compound **1**. For the generation of compound **1**, in vivo and in vitro data were consistent (Fig. 2a).

Surprisingly, five additional compounds, **2**–**6**, were detected in this fermentation (Fig. 2a, Supplementary Fig. 3). Compounds **2**–**4** (Fig. 1) were purified from *E. coli* GB230 fermentation and their structures were determined using NMR spectroscopy. Compound **1** was confirmed as 7-epi-α-selinene^18^, a widely used sesquiterpene in the field of perfumery and flavoring. Compound **2** and the main product **3** were confirmed to be 1-(3-methyl-2-butenyl)-indole and 3-(3-methyl-2-butenyl)-indole, respectively, in which a dimethylallyl moiety derived from DMAPP is substituted at the indole N1 and C3 sites, respectively. Compound **4** (Supplementary Table 1) contains two prenyl groups at both C3 and N3 and was confirmed as 1,3-bis(3-methyl-2-butenyl)-indole, which was previously produced by chemical synthesis and can be used as the precursor for the production of cholinesterase inhibitor debromoflustramine B^19^. Thus, in addition to being a terpene cyclase, AaTPS is an indole prenyltransferase evolutionarily unrelated to indole prenyltransferases that adopt the ABBA fold^20–22^.

### Relationship of indole and polyisoprenoid precursors

The accumulation of IPP, DMAPP, and other polyisoprenoid diphosphates is toxic to *E. coli*^23–25^. Indole is recognized as the substrate for tryptophan synthase (TrpA and TrpB) to produce tryptophan for protein biosynthesis. Additionally, the concentrations of indole and terpene are negatively correlated in a taxadiene overexpression system^26^, which implies that there is negative regulation between them. To explore the regulatory mechanism, three *E. coli* strains were studied: (1) *E. coli* BL21 (DE3) was utilized as the negative control; (2) mutant *E. coli* C1 was used due to its enhanced mevalonate pathway which in turn enhances IPP/DMAPP generation; and (3) *E. coli* GB230 was constructed for the overproduction of AaTPS sesquiterpene products by overexpressing Idi, FPPS, and AaTPS in *E. coli* C1.

**Fig. 3:**
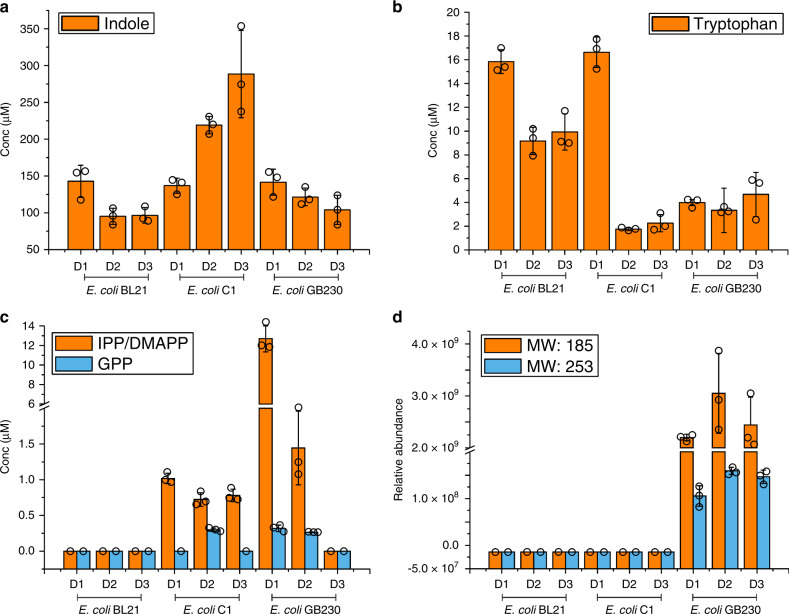
Accumulation of compounds in engineered *E. coli* strains. Plates **a**–**d** represent the accumulation of (**a**) indole, (**b**) tryptophan, (**c**) IPP/DMAPP and GPP, and (**d**) prenylindoles in *E. coli* BL21(DE3), *E. coli* C1 (with overexpressed heterologous mevalonate pathway), and *E. coli* GB230 (with Idi, FPPS and AaTPS overexpressed in *E. coli* C1). Cells were harvested at 24, 48, and 72 h after IPTG induction. Cells of each engineered strain were harvested (5 × 10^6^ cells, ~0.02 g dry cell weight) and extracted with 300 μL extraction solution (methanol:acetonitrile:water = 2:2:1). The organic layer was analyzed by LC-MS/MS and product quantities were averaged for four independent experiments. Conc represents the concentration of compounds detected in *E. coli*. Error bars indicate the standard deviations of three biological replicates. Source data are provided as a Source Data file.

To evaluate the accumulation of indole, tryptophan, polyisoprenoid precursors and the resulting prenylindoles in the mutants, cells were harvested at 24, 48, and 72 h after IPTG induction. Of each engineered strain, 5 × 10^6^ cells (~0.02 g dry cell weight) were harvested and extracted with 300 μL solvent mixture (methanol:acetonitrile:water = 2:2:1). The cellular concentrations of targeted compounds were determined using a mass spectrometry-based relative quantification method. The resulting data showed that the concentration of indole is positively correlated with isoprenoid precursors and negatively correlated with tryptophan (Fig. 3a–c). Compared with the negative control (*E. coli* BL21), *E. coli* C1 with its enhanced IPP/DMAPP pathway significantly increases the concentration of indole; this phenomenon is alleviated when the downstream sesquiterpene forming pathway is introduced in *E. coli* GB230 (Fig. 3a). Prenylindoles were detected in *E. coli* GB230 with mono-DMAPP substituted prenylindole (molecular weight, MW = 185) as the main product (Fig. 3d). The concentrations of indole and isoprenoid precursors decreased together when the terpene and prenylindoles were produced (Fig. 3a, c, d).

We suggest that the indole prenyltransferase activity here serves as a regulatory mechanism to alleviate cellular toxicity that would otherwise result from the accumulation of isoprenoid diphosphates. An enhanced mevalonate pathway results in the accumulation of IPP/DMAPP, which likely inhibits the activity of tryptophan synthase (TrpB) and hence inhibits the metabolic flux of indole for tryptophan biosynthesis. When the downstream terpene production pathway is operative, IPP/DMAPP are utilized as precursors for terpene biosynthesis, thereby reducing the inhibition of TrpB and consequently reducing cellular indole concentrations. In other words, when the catalytic efficiency of terpene biosynthesis is low, IPP/DMAPP accumulate to excess, which is toxic for the cell. In response to toxic levels of IPP/DMAPP, the cryptic aromatic prenyltransferase activity of the terpene cyclase is activated to utilize excess DMAPP and indole to generate mono- and di-substituted prenylindoles. Consequently, indole and DMAPP levels diminish to mitigate cytotoxicity.

### In vitro and kinetic assays of AaTPS

To systematically evaluate the activity of AaTPS, in vitro assays of temperature, pH, and metal-ion dependence were performed. Most interestingly, terpene synthase and indole prenyltransferase activities can be switched by different pH values (Fig. 2d). Optimal activities for terpene synthase and indole prenyltransferase were observed at pH 7.1 and pH 8.6, respectively. These results suggest that the terpene cyclase activity is favored in a neutral environment and indole prenyltransferase activity is favored under alkaline conditions. The optimal temperature for both functions was 28 °C (Fig. 2e). The metal-ion dependence assay showed that, in contrast with traditional fungal indole prenyltransferases which do not contain signature metal-binding motifs^27^, both activities of AaTPS required Mg^2+^ to generate the resulting sesquiterpene and prenylindole compounds. Given that substrate promiscuity is commonly observed for aromatic prenyltransferases^28^, we used DMAPP, GPP, and FPP as potential prenyl group donors, and used indole and its analogues (**15***–***25**) as acceptors to investigate substrate promiscuity of AaTPS. The resulting data show that AaTPS requires DMAPP as an exclusive prenyl donor but can utilize indole and compounds **22**–**24** to generate a series of prenylated products **26**–**30** (Supplementary Figs 4 and 5). The steady-state kinetics values of AaTPS for cyclization and aromatic prenylation activities revealed that the catalytic efficiency (*k*_cat_/*K*_M_) of cyclase activity (*K*_M_ = 25.2 μM, *k*_cat_ = 3.6 s^−1^, and *k*_cat_/*K*_M_ = 0.14 s^−1^ μM^−1^ for FPP) is 5000 times higher than that of aromatic prenyltransferase activity (*K*_M_ = 125.3 μM, *k*_cat_ = 3.5 × 10^−3^ s^−1^, and *k*_cat_/*K*_M_ = 2.8 × 10^−5^ s^−1^μM^−1^ for indole) (Supplementary Table 2). The results of these experiments confirm that AaTPS can function as a sesquiterpene cyclase and an indole prenyltransferase, although the prenyltransferase activity is much lower than native cyclization activity.

### Structures of bifunctional AaTPS

**Fig. 4:**
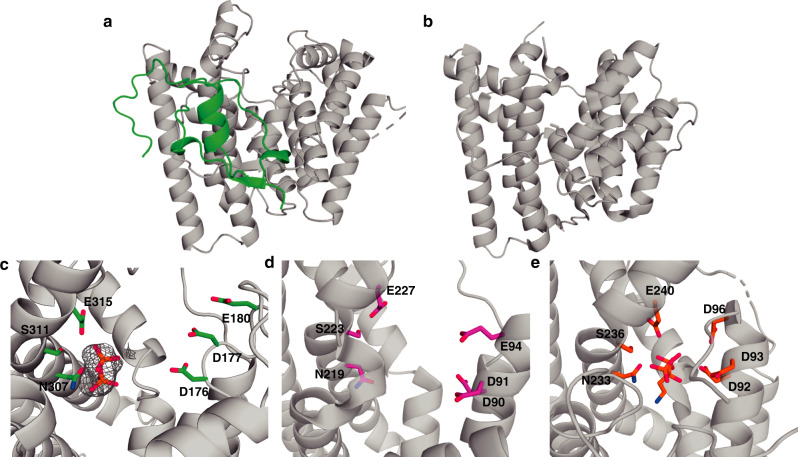
Structural comparison of AaTPS and other terpene synthases. **a** Overall structure of AaTPS. The additional N-terminal segment is green. **b** Overall structure of aristolochene synthase from *Aspergillus terreus* (PDBID 2OA6). **c**–**e** Comparison of the DDXXD/E and NSE/DTE motifs. **c** Omit map of inorganic pyrophosphate in the active site of AaTPS, contoured at 4.5σ. **d** Close-up view of the active site of aristolochene synthase from *Aspergillus terreus* (PDB 2OA6). **e** Active site of the cyclization domain of fusicoccadiene synthase (PDB 5ERM).

To understand the bifunctionality of AaTPS, we solved the crystal structures of AaTPS in its apo-form and complexed with inorganic pyrophosphate (PP_i_) at 2.6 Å and 2.7 Å resolution, respectively. The overall structure of AaTPS exhibits the characteristic α-fold of a class I terpene cyclase. The overall structure of AaTPS is homologous to class I terpene synthases (root-mean-square (rms) deviation of 2.9 Å for 169 Cα atoms of aristolochene synthase from *Aspergillus terreus* (PDBID: 2OA6)^15^, sequence identity 21%; 2.8 Å for 177 Cα atoms of the cyclization domain of fusicoccadiene synthase from *Phomopsis amygdali* (PDBID: 5ERM)^29^, sequence identity 19%). The comparison of AaTPS with aristolochene synthase (ATAS) from *A. terreus* reveals that AaTPS possesses an additional loop structure at its N-terminal region (~85 residues). Enzyme activity and solubility of AaTPS decrease when we truncate 60 residues from the N-terminus. Furthermore, the DDXXD and NSE/DTE metal-binding motifs are conserved (Fig. 4).

The crystal structure of the AaTPS–PP_i_ complex provides important mechanistic information, in that the PP_i_ anion interacts through Mg^2+^ coordination with the metal-binding motifs. The diphosphate group of DMAPP should do the same, with the prenyl moiety binding in the hydrophobic active site cavity as shown for substrate binding to aristolochene synthase^15^. Indole must bind adjacent to the prenyl moiety to enable carbon–carbon bond formation following metal-triggered substrate ionization.

Aromatic residues typically define part of the active site contour in a terpene cyclase active site and influence cyclization fidelity and specificity. To probe the influence of active site aromatic residues on the cyclase and aromatic prenyltransferase activities of AaTPS, we prepared the F146A, F244A, and Y272A mutants (Supplementary Fig. 6) and measured the steady-state kinetic parameters for FPP cyclization and indole prenylation (Supplementary Tables 2 and 3 and Supplementary Fig. 7). Apart from a modest three-fold increase in turnover number (*k*_cat_) observed for FPP cyclization catalyzed by F146A AaTPS, turnover numbers and catalytic efficiencies (*k*_cat_/*K*_M_) do not vary substantially. These results indicate that expansion of the hydrophobic cavity by the large-to-small amino acid substitutions do not compromise catalysis of FPP cyclization or indole prenylation.

While the three-fold increase in *k*_cat_ observed for FPP cyclization catalyzed by F146A AaTPS is modest, it is instructive to take a closer look at this mutant. The three-fold increase in *k*_cat_ is offset by a nearly three-fold increase in Michaelis constant *K*_M_, so that the catalytic efficiency (*k*_cat_/*K*_M_) of this mutant is comparable to that of the wild-type enzyme (Supplementary Table 2). Interpretation of such relatively small changes in steady-state kinetic parameters must be made cautiously. However, we speculate that since F146 is closest to the substrate diphosphate binding site (Supplementary Fig. 6), the widening of the active site resulting from the F146A substitution slightly decreases enzyme–substrate contact in the Michaelis complex, resulting in a slight increase in *K*_M_. Additionally, slight decreases in enzyme–substrate or enzyme–product contact may slightly increase *k*_cat_ by enhancing product release, which is known to be the rate-limiting step for catalysis by sesquiterpene cyclases^30,31^.

Even though catalytic efficiency is relatively invariant among the AaTPS mutants studied, it is interesting that product profiles for the indole prenylation reaction differed from those of the wild-type enzyme (Supplementary Fig. 8). These results suggest that the three-dimensional contour of the hydrophobic active site is important for directing the aromatic prenyltransferase reaction, consistent with the binding of the aromatic prenyl acceptors in the lower section of the cavity away from the PP_i_ binding site. An active site contour modified by mutagenesis will allow the indole co-substrate to bind with an alternative orientation, which will lead to the generation of an alternative prenylindole product. These results are consistent with mutagenesis studies of the active site contours of other sesquiterpene cyclases, where even single amino acid substitutions can dramatically alter the cyclization product formed^32–34^. Here, too, as long as the critical metal-binding motifs are not perturbed, mutant enzymes can exhibit reaction kinetics comparable to those of wild-type enzymes.

### Universal prenyltransferase activity of terpene synthases

The similarity of the active site architectures among AaTPS and other terpene synthases suggests that the aromatic prenyltransferase activity discovered for AaTPS might be a ubiquitous phenomenon in the greater family of class I terpene cyclases. To explore this possibility, the terpene cyclases FgFS, FgJ01056 (longiborneol synthase from *F. graminearum*), FgJ09920 (koraiol synthase from *F. graminearum*), FgGS, FgMS, and CgDS, which had been previously characterized in our lab^11,13,14,35^, and the plant-derived taxadiene synthase (TXS)^26^ were selected for in vitro assay. The resulting data reveal that when incubated with DMAPP and indole, all of these terpene cyclases except FgMS produced prenylindoles **5**–**10** (Fig. 2b, c). Interestingly, the diterpene synthase FgGS can accept GPP as substrate donor to produce long-chain prenylindoles **11**–**14** (Fig. 2c, Supplementary Fig. 3). Due to low catalytic efficiency in the generation of compounds **11** and **14**, the structures of these compounds were characterized by comparison with chemically synthesized prenylindoles. Compounds **11** and **14** were characterized as previously reported (E)-1-(3,7-dimethylocta-2,6-dien-1-yl)-1H-indole and (E)-3-(3,7-dimethylocta-2,6-dien-1-yl)-1H-indole, respectively^36,37^. This characterization implies that the active site cavity of FgGS is sufficiently large to allow the binding of indole as well as C_5_ DMAPP or C_10_ GPP to produce prenylindoles. Since production of farnesylated indoles is not observed, the FgGS active site is not sufficiently large to accommodate and activate the C_15_ substrate. Based on our discovery that a wide variety of terpene cyclases exhibit aromatic prenyltransferase activity, we suggest that terpene cyclases may have a universal cryptic function as indole prenyltransferases.

### Structure and mechanism of bifunctional FgGS

To determine structure–function relationships in a second terpene cyclase with cryptic aromatic prenyltransferase activity, we determined the X-ray crystal structure of FgGS complexed with 3 Mg^2+^ ions, PP_i_, and the benzyltriethylammonium cation (BTAC) at 1.46 Å resolution. FgGS also adopts the canonical α fold of a class I terpene cyclase (Supplementary Fig. 9a) and is most similar to that of the cyclization domain of fusicoccadiene synthase^29^ (32% amino acid sequence identity^5^; rms deviation = 1.2 Å for 213 Cα atoms (Supplementary Fig. 9b).

The 3 Mg^2+^ ions are coordinated by the aspartate rich and NSE metal-binding motifs, PP_i_, and water molecules to complete octahedral metal coordination polyhedra (Supplementary Fig. 9c), as observed in other high resolution structures of ligand-bound class I terpene cyclases^3,38^. Typically, the aspartate-rich motif appears as DDXX(D/E). However, the second aspartate residue in the aspartate-rich motif of FgGS is substituted with serine, so this motif appears as D^94^SVLE^98^. Even so, metal coordination interactions are as expected: the side chain of D94 coordinates to Mg^2+^_A_ and Mg^2+^_C_ with *syn*–*syn* bidentate geometry, and Oδ2 of E98 coordinates to Mg^2+^_A_ and Mg^2+^_C_ with monodentate bridging geometry. Interestingly, Oδ1 of E98 coordinates to a Na^+^ ion outside of the enclosed active site cavity. It is unusual to see additional metal ions bound near a class I terpene cyclase active site, and even more so involving a ligand to a catalytically obligatory Mg^2+^ ion. The NSE metal-binding motif appears as N^209^DYFSFDRE^217^ and chelates Mg^2+^_B_.

The PP_i_ anion is stabilized by hydrogen bonds with R165, T169, N209, R216, R300, and Y301. These hydrogen bonds, as well as metal coordination interactions, are believed to activate the substrate diphosphate group in the first step of catalysis to generate the reactive allylic cation and PP_i_.

**Fig. 5:**
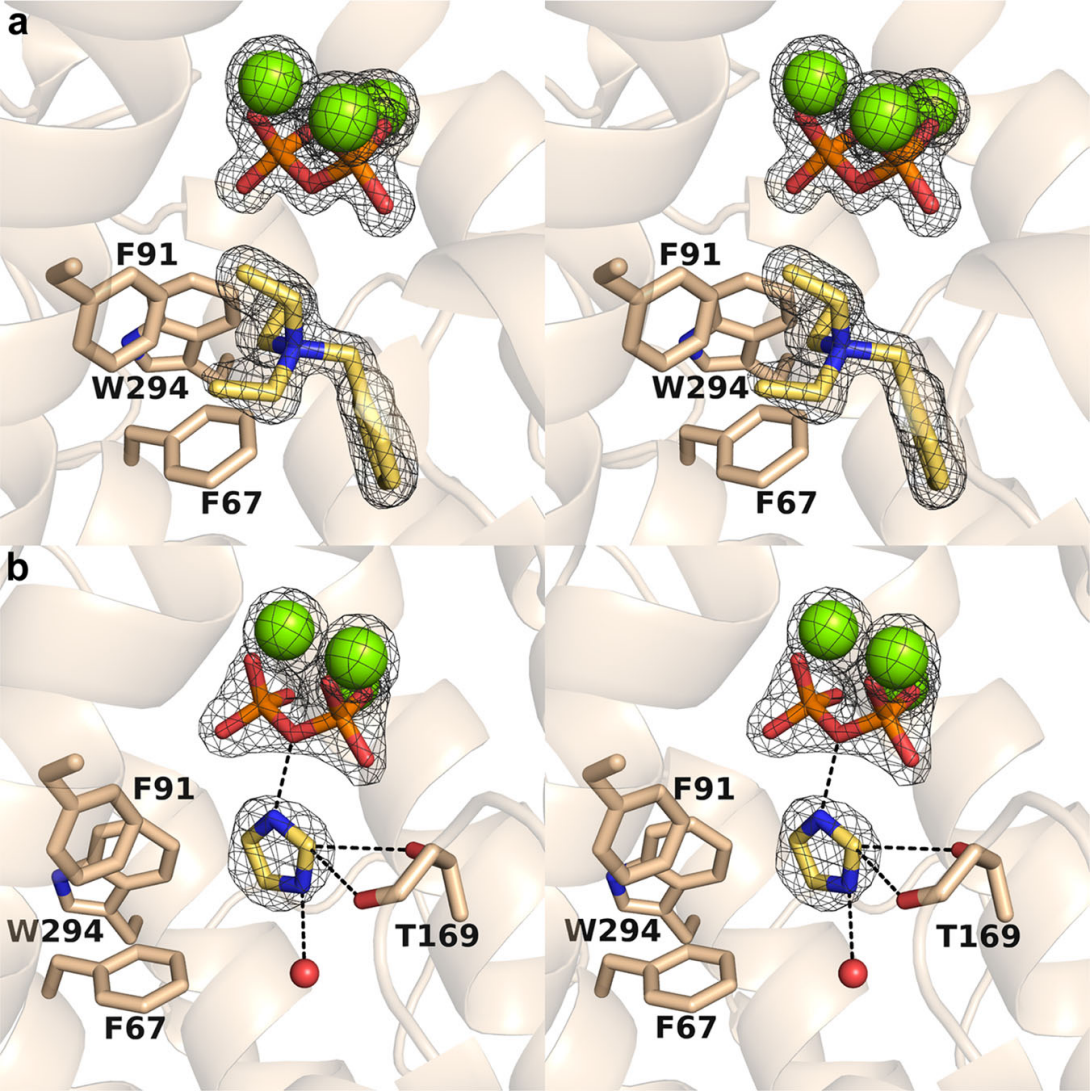
Binding of ligands in the active site of FgGS. **a** Stereoview of Polder omit maps of Mg^2+^ ions (contoured at 9.0σ), PP_i_(contoured at 16.0σ), and BTAC (contoured at 6.5σ) in the FgGS–Mg^2+^_3_–PP_i_–BTAC complex. **b** Stereoview of Polder omit maps of Mg^2+^ ions (contoured at 5.0σ), PP_i_(contoured at 7.0σ), and imidazole (contoured at 6.0σ) in the FgGS–Mg^2+^_3_–PP_i_–imidazole complex, with hydrogen bonds indicated by dashed black lines.

The active site contour is distinctly shaped with two sections, an upper and lower cavity as also observed for AaTPS. The BTAC cation binds in the lower cavity and its positively charged quaternary amino group engages in cation-π interactions with F67, F91, and W294 (Fig. 5a). Additionally, the aromatic ring of BTAC makes a favorable edge-to-face interaction with F67.

Terpene cyclases manage and manipulate high energy carbocation intermediates in isoprenoid cyclization cascades, and Mg^2+^_3_-PP_i_ binding typically triggers complete active site closure to protect carbocation intermediates from being quenched by bulk solvent. However, active site closure in FgGS also traps two ordered water molecules at the periphery of the lower active site cavity. One water molecule (W10) hydrogen bonds with the side chains of N209, N291, and S295. The second water molecule (W15) hydrogen bonds with the backbone carbonyl of A171 and the side chain of Q287. Additionally, in the upper active site cavity there are two ordered waters (W21 and W22) and an ethylene glycol molecule (monomer A), or five ordered water molecules (W93, W101, W126, W222, and W263) and an ethylene glycol molecule (monomer B). Given that FgGS generates hydroxylated products^13^, it is possible that these trapped water molecules are capable of quenching carbocation intermediates in catalysis.

The crystal structure of FgGS complexed with Mg^2+^_3_-PP_i_ and imidazole was determined at 2.15 Å resolution. The protein structure in this complex is essentially identical to that in the BTAC complex (rms deviation = 0.15 Å for 292 Cα atoms in monomer A). Metal coordination and hydrogen bond interactions for the PP_i_ group in this complex are identical to those observed in the BTAC complex. Unexpectedly, imidazole and not indole is trapped in the active site of this structure (Fig. 5b). Imidazole was required for crystallization and the concentrations of imidazole and indole were 50 mM and 1 mM, respectively. Therefore, imidazole out-competed indole for binding in the active site during crystallization.

Intermolecular interactions suggest that imidazole is bound as the positively charged imidazolium cation. While it is possible that the imidazolium cation can adopt other orientations, the orientation modeled in the crystal structure was selected to optimize hydrogen bond interactions in the active site. The imidazolium N2-H group donates a hydrogen bond to the bridging oxygen of PP_i_, and the imidazolium N1-H group donates a hydrogen bond to a water molecule (monomer A) or ethylene glycol (monomer B). Due to the electron withdrawing nature of the flanking nitrogen atoms, the acidity of the imidazolium C1-H proton is enhanced and it serves as a bifurcated hydrogen bond donor to the side chain and main chain of T169. A glycerol molecule from the cryoprotectant solution binds adjacent to the imidazolium cation.

**Fig. 6:**
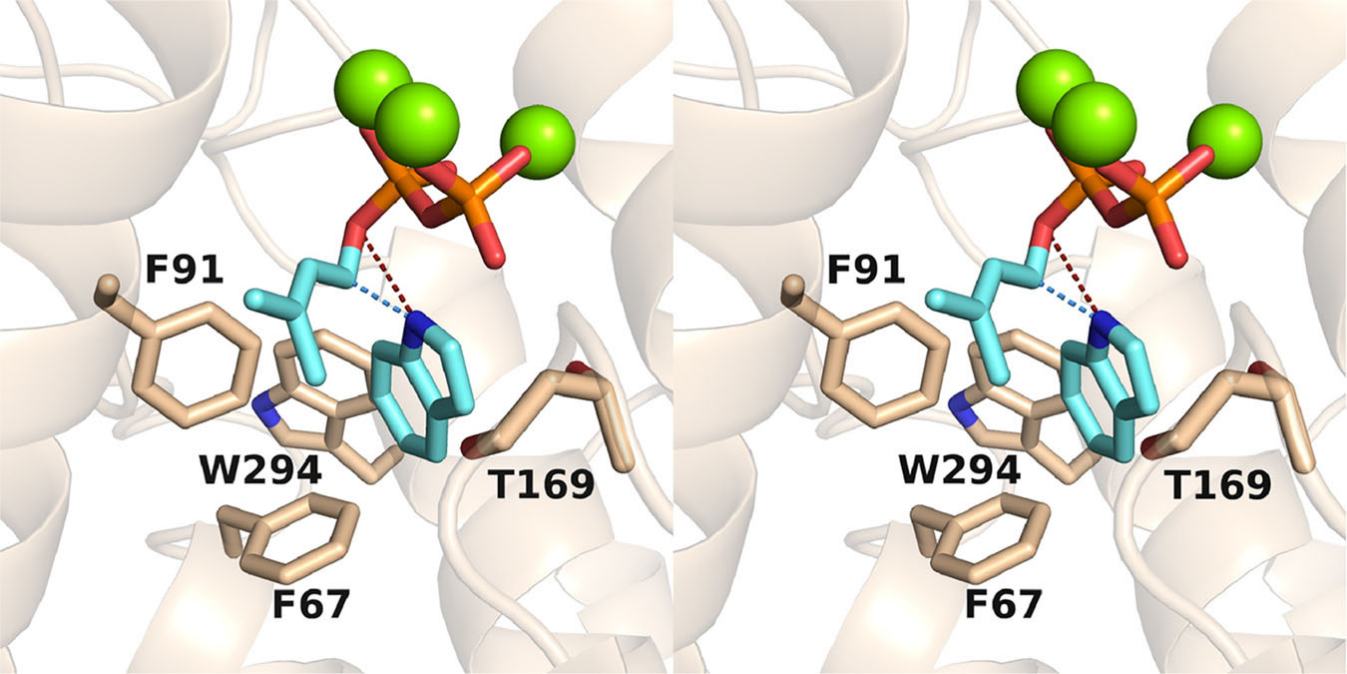
Model of the FgGS–Mg^2+^_3_–DMAPP–indole complex. In this stereoview, the position of the diphosphate group of DMAPP corresponds to the experimentally observed position of the PP_i_ group, and the position of the 5-membered ring of indole corresponds to experimentally observed position of the imidazole group in Fig. 5b. A light blue dashed line indicates the trajectory of bond formation between C1 of DMAPP and N1 of indole. If indole were flipped, this trajectory would correspond to bond formation between C1 of DMAPP and C3 of indole. A dark red dashed line indicates the most probable fate of the indole proton ultimately abstracted by co-product inorganic pyrophosphate.

It is interesting to speculate that the binding of imidazole represents a binding site for indole in the prenyltransferase reaction, and the glycerol molecule bound adjacent to imidazole may represent a binding site for the dimethylallyl moiety of DMAPP. Accordingly, a model of the ternary complex between FgGS, DMAPP, and indole shows that either the N1 or the C3 atom of indole (depending on the binding orientation of indole) will be adjacent to the C1 atom of the allylic cation resulting from DMAPP ionization (Fig. 6).

While all side chains of both complexes are in approximately the same position in the active site, R216 of the FgGS–Mg^2+^_3_–PP_i_–imidazole complex in monomer A adopts two conformations. In the in conformation, R216 donates hydrogen bonds to PP_i_ and a Mg^2+^_A_-bound water molecule; in the out conformation, R216 donates a hydrogen bond to the backbone carbonyl of Y301. Since Y301 also donates a hydrogen bond to the bound PP_i_ anion, it is possible that the formation of a hydrogen bond between Y301 and PP_i_ initiates active site closure. Hydrogen bond switching from R216-Y301 to R216-PP_i_-Y301 may be a key feature of active site closure in FgGS.

## Discussion

Catalytic versatility is an inherent property of many terpene cyclases, in that they can often generate multiple cyclization products. However, we have now shown that additional biosynthetic potential of these enzymes can be unmasked when they are studied under a wider range of physiological conditions with a variety of substrates. Here we report that by taking advantage of the efficient sesquiterpene overproduction chassis *E. coli* C1, cryptic aromatic prenyltransferase activity has been discovered in a class I terpene cyclase. Aromatic prenyltransferase activity was confirmed by in vitro and in vivo assays of AaTPS. The universality of this cryptic feature was confirmed by in vitro assays of other class I terpene cyclases such as FgGS, thereby revealing another approach for the production of prenylindoles. Further investigation of the aromatic prenylation activity of terpene cyclases from various organisms will reveal how broadly this cryptic function is distributed in nature.

It should be noted that bifunctional chimeric terpene synthases have already been identified in fungal species, but these enzymes contain two different active sites—a prenyl diphosphate synthase domain and a class I or II terpene cyclase domain connected by a long linker peptide^3^. In contrast, the class I terpene cyclases described herein contain just one active site as confirmed by the crystal structures of AaTPS and FgGS. The terpene cyclization and aromatic prenylation reactions catalyzed by these bifunctional enzymes occur in the same active site.

Such bifunctionality in a single enzyme active site is reminiscent of the flavin prenyltransferase UbiX, which catalyzes both prenylation and cyclization reactions in ubiquinone biosynthesis^39,40^. This enzyme utilizes DMAP(P) and flavin as substrates to catalyze the prenylation of the N5 position of flavin with a dimethylallyl moiety; Friedel–Crafts alkylation of the flavin C6 atom then yields the tetracyclic flavinoid prFMNH_2_. Here, too, sequential reactions are catalyzed in the same enzyme active site. However, UbiX exhibits no sequence or structural similarity to class I terpene cyclases.

Notably, the structure of another enzyme of ubiquinone biosynthesis, UbiA, yields mechanistic clues regarding the aromatic prenylation activity of class I terpene cyclases. UbiA is a membrane-embedded protein that shares the α fold of a class I terpene cyclase yet catalyzes an aromatic prenylation reaction initiated by metal-triggered ionization of the prenyl diphosphate co-substrate^4,7^. UbiA possesses two aspartate-rich motifs, NDXXDXXXD and DXXXD, which are evolutionarily related to the characteristic metal-binding motifs of class I terpene cyclases^3,38^.

In view of the structural similarity between class I terpene cyclases and UbiA-type aromatic prenyltransferases, the crystal structures of AaTPS and FgGS provide important mechanistic inferences on the aromatic prenylation reactions catalyzed by these terpene cyclases. First, the binding site of the substrate diphosphate group is established by the binding of the PP_i_ anion in the active sites of AaTPS and FgGS (Figs. 4c and 5a). Numerous structural studies of other terpene cyclases with bound substrate analogues show that the binding modes of the substrate diphosphate group and the PP_i_ coproduct are identical^3^. In the case of substrate binding, the diphosphate group coordinates to 3 Mg^2+^ ions and the lipophilic tail binds deep in the hydrophobic active site pocket^15^. Similarly, the diphosphate group of DMAPP coordinates to 3 Mg^2+^ ions and its isoprenoid tail extends into the hydrophobic active site pocket, as exemplified by the structure of FgGS complexed with PP_i_ and BTAC (Fig. 5a). The binding of the bulky BTAC molecule reveals that there is more than enough volume to accommodate the small 5-carbon prenyl group of DMAPP in the active site pocket. Indeed, there would be sufficient volume to allow for the binding of indole in the pocket alongside the prenyl group of DMAPP.

The crystal structure of FgGS complexed with the PP_i_ anion and imidazole shows how a heteroaromatic group can bind in the active site pocket (Fig. 5b). Indeed, the binding of indole can be modeled after the binding mode of imidazole, with the 5-membered imidazole ring establishing the binding site of the 5-membered ring of indole. With the binding of DMAPP modeled after the binding of the PP_i_ anion, both substrates can be accommodated easily in the FgGS active site in orientations that support the trajectories of bond-breaking and bond-forming steps in the prenylation reaction (Fig. 6).

**Fig. 7:**
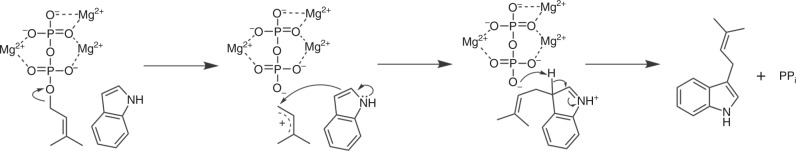
Proposed mechanism of indole prenylation. The general reaction mechanism of aromatic prenylation catalyzed by class I terpene cyclases is shown. If the indole were flipped, then N-prenylation would occur. The position of indole in the active site pocket is based on the three-dimensional model in Fig. 6, which in turn is based on the experimentally determined position of imidazole in Fig. 5b.

Given the parallels between class I terpene cyclases and UbiA-type aromatic prenyltransferases^4,7^, our structural data suggest that their aromatic prenylation mechanisms are similar (Fig. 7). First, substrate binding results in active site closure to protect carbocation intermediates from premature quenching by bulk solvent. Ionization of the DMAPP diphosphate group triggered by metal coordination then yields an allylic cation and a Mg^2+^_3_–PP_i_ complex identical to that observed bound to FgGS in Fig. 5. The allylic cation is capable of alkylating the adjacent heteroaromatic indole ring, the binding site of which is indicated by the binding of the heteroaromatic imidazole moiety (Fig. 5b). Following carbon–carbon (or carbon–nitrogen) bond formation, the acidic proton of the indole carbocation intermediate is abstracted by the PP_i_ co-product, as proposed for UbiA^7^, to yield the prenylindole product.

Isoprenoid precursors DMAPP, IPP, and GPP are toxic when they accumulate in *E. coli*^23–25^, and Ajikumar reported that production of indoles and taxadiene is negatively correlated and indole is easily accumulated when the supply of isoprenoid precursors overwhelms substrate availability of taxadiene synthase^26^. Therefore, we suggest that, just like the effect of flood discharge, terpene synthases might utilize aromatic compounds together with DMAPP or GPP as substrates to produce prenylindoles to alleviate the toxicity that would otherwise result from the accumulation of aberrantly high cellular concentrations of isoprenoid precursors. Of course, further proof is necessary to establish that the cryptic prenyltransferase activity has truly protective function in a wider array of species.

In conclusion, our in vivo and in vitro analyses and structural studies unveiled the cryptic aromatic prenylation activity of class I terpene cyclases. Further structural and functional comparisons of terpene cyclases with other enzymes of terpenoid biosynthesis may reveal additional insight regarding the evolution of catalysis in these systems.

## Methods

### General materials

All reagents commercially supplied were used as received. *E. coli* BL21 (DE3) *F*^−^*dcmompThsdSB(rB*^−^*mB*^−^*)gal* was obtained from Invitrogen (Carlsbad, CA, USA). *Alternaria alternata* TPF6 was isolated from *Taxus chinensis* and cultivated in potato dextrose agar (PDA) media^17^. *E. coli* XL1-blue was used for plasmid construction in this study. Phusion High-Fidelity polymerase was used for gene amplification. T4 DNA ligase and DNA restriction enzymes used in plasmid construction were purchased from Thermo (Thermo Scientific, Waltham, USA). The sequence of AaTPS was codon-optimized and synthesized by GeneScript (GenScript USA Inc., Piscataway, NJ, USA). The RNeasy Plant Mini kit and RNase-Free DNase Set were from Qiagen (Valencia, CA, USA). The PrimeScript reagent kit and SYBR Premix Ex Taq II were from TaKaRa (Kyoto, Japan). Strain and plasmids are summarized in Supplementary Table 4, the primers used in this study are listed in Supplementary Table 5, and the sequence of terpene synthases used in this study is shown in the Supplementary Dataset 1.

### Evolution of enzyme families

To investigate the phylogenetic relationship of terpene cyclases with soluble aromatic prenyltransferases in fungi, multiple sequence alignments were performed using Clustal W 2.0.12. The Maximum Likelihood approach based on the Poisson correction model was employed to determine evolutionary history^41^. The phylogenetic tree shown in Supplementary Fig. 1 yielded the highest log likelihood (−12491.93) in this analysis. Also indicated in this figure are percentages of trees in which associated taxa are clustered. The Neighbor-Join and BioNJ algorithms were applied to a matrix of pairwise distances estimated using a JTT model to establish the initial tree for the heuristic search. The topology with the superior log likelihood value was then selected. The tree shown in Suppementary Fig. 1 is drawn to scale; branch lengths were determined through the number of substitutions per site in an analysis of 18 different amino acid sequences. A total of 266 positions are contained in the final dataset, from which positions containing gaps and missing data were omitted. MEGA 7 software, including a Maximum Likelihood algorithm, was utilized for these evolutionary analyses (ref. ^42^). The resulting data showed that terpene cyclases and soluble aromatic prenyltransferases included in this study were clustered into two groups, indicating an independent evolutionary relationship between these two enzyme families.

### Construction of AaTPS expression and fermentation plasmids

The genomic DNA sequence of AaTPS was amplified from *A. alternata* TPF6, using the primer pair P1/P2 and subcloned into pET28a to generate pET28a-AaTPS (all primers are listed in Supplementary Table 5). The His tag ligated AaTPS was subsequently amplified with primer pair P3/P4, and the backbone of pGB94 was amplified with P5/P6; these two fragments were then digested with XbaI/BstEII and assembled to generate pGB171. After verifying the coding sequence, AaTPS was codon optimized, amplified by primer pairs P7/P8 and P7/P9, and subcloned into pET28a and pET21a to generate pGB163 and pGB228, respectively. The XbaI/XhoI digested fragment (FPPS-Idi) of pGB308^13^ was then inserted into the SpeI/XhoI digested plasmids pGB228 to generate pGB230. For the production of sesterterpene produced by AaTPS, pGB230 was transformed into *E. coli* C1 to generate *E. coli* GB230 (harboring pMH1, pFZ81, and pGB230).

### Activation and transcription of AaTPS in A. alternata TPF6

*Agrobacterium*-mediated transformation of *A. alternata* TPF6 was carried out according to the previously described method^17^. Spores of *A. alternata* TPF6 were cultivated for 28 days on PDA slants at 28 °C, then harvested and washed with sterile distilled water. The *A. tumefaciens* EHA105 transformed with plasmid pGB171 in LB medium was incubated overnight at 28 °C, then cultivated for an additional 6 h following dilution with induction medium to OD_600_ = 0.15. Spores of *A. alternata* TPF6 were diluted with *A. tumefaciens* EHA105 at a concentration of 10^6^ per liter. Spores were plated onto sterile cellophane and cultivated at 25 °C for 3 days over co-cultivation medium. Following cultivation, the cellophane was transferred to a PDA plate and cultivated at 28 °C with 50 mg L^−1^ hygromycin B and 500 mg L^−1^ cephalosporin. Transformant colonies were selected on PDA plates containing 200 mg L^−1^ of hygromycin B and verified by PCR amplification. Finally, mutant *A. alternata* GB171 with AaTPS under the control of the alcA promoter was acquired.

To verify the transcription of AaTPS, the total RNA was extracted from *A. alternata* GB171 and cDNA was synthesized using a PrimeScript reagent kit. Subsequently, the coding sequence of AaTPS was amplified from the cDNA library of *A. alternata* GB171 and subcloned into pMD18-T for sequencing. After verification of the coding sequence, AaTPS was codon optimized for expression and fermentation in *E. coli*.

To reveal the regulatory mechanism for indole and terpene production, cells of *E. coli* BL21 (DE3), *E. coli* C1, and *E. coli* GB230 were harvested at 24, 48, and 72 h after IPTG induction. 5 × 10^6^ cells (~0.02 g dry cell weight) were extracted with 300 μL of extraction solvent (methanol:acetonitrile:water = 2:2:1) twice. The sample supernatant was used for qualitative analysis. Each sample supernatant was used for quantitative analysis.

We established two methods to analyze intermediates using Ultimate 3000 HPLC system coupled with Q Exactive MS (Thermo Scientific). The absolute concentrations of IPP/DMAPP, GPP, indole, and tryptophan were calculated using standard curves with standardized solutions. Peak area was used for relative concentrations of the products resulting in MW 185 and MW 253 due to a lack of standards.

For detection of IPP/DMAPP and tryptophan, ZIC-pHILIC column (100 mm × 4.6 mm i.d., 5 μm; Merck) was utilized for compounds separation at 25 °C. Solvent A: 5% aqueous acetonitrile with 15 mM ammonium bicarbonate (pH = 8.5) and solvent B: acetonitrile used as mobile phase at a flow rate of 0.2 mL min^−1^. Sample tray temperature was set as 4 °C. Gradient program was as follows: 90% solvent B (0–1 min), 90–30% solvent B (1–13 min), 30% solvent B (13–16 min), 30–90% solvent B (16–17 min), 90% solvent B (17–25 min). Electrospray ionization was utilized in negative mode for detection. Ion source parameters were as follows: sheath gas 30 arb, aux gas 5 arb, spray voltage 3.1 kV, capillary temp. 320 °C, S-lens RF level 50 kV, Aux gas heater temp. 250 °C. Full scan MS mode was used for quantitative analysis and full scan MS/dd-MS2 (full scan mode plus targeted parent ions MS2) mode was set for qualitative analysis. Detailed parameters of MS were as follows: scan range 100–1000 *M/Z*, resolution 70,000 for full MS, resolution 17,500 for full MS/dd-MS2, isolation window 1.4 *M/Z*, NCE30.

For detection of GPP, indole, MW 185, and MW 253, ACE Ultra Core 2.5 Super c18 column (100 mm × 2.1 mm i.d., 2.5 um) was used and samples were eluted at 35 °C. Mobile phase A was 5 mM ammonium bicarbonate in water (pH 9.5) and mobile phase B was acetonitrile. Flow rate was set 0.2 mL min^−1^. Sample tray temperature was set as 4 °C. Gradient program was as follows: 5% solvent B (0 min), 5–90% solvent B (0–10 min), 90% solvent B (10–11 min), 90–5% solvent B (11–11.1 min), 5% solvent B (11.1–15 min). Ion source and MS conditions were the same as IPP/DMAPP detection.

### Protein expression and purification

Plasmids for protein expression were transformed into *E. coli* BL21 (DE3). Cells containing plasmid were cultivated in 2-L flasks containing 1 L LB medium at 37 °C with 50 mg L^−1^ kanamycin. When the OD_600_ reached 0.6–0.8, 0.1 mM IPTG was added to the cultures, which were then grown for an additional 24 h at 16 °C. The cells were harvested and then resuspended in 40 mL buffer A [50 mM Tris-Cl (pH 7.6), 300 mM NaCl, 4 mM β-mercaptoethanol].

The cell suspensions were lysed using a high-pressure homogenizer and centrifuged at 30,000*g* for 45 min. The supernatant was then filtered, and 6% buffer B (500 mM imidazole in buffer A) was added to adjust the supernatant to 30 mM imidazole to reduce nonspecific binding. Biologic DuoFlow Chromatography System (Bio-Rad) was used for protein purification. The His-tagged protein was purified using a Ni-NTA column (GE Healthcare Life Science, USA) according to the methods described by Zhu^10^. The protein was then concentrated and preserved in PB buffer (100 mM phosphate buffer with 10% glycerol) for in vitro assays to test the catalysis for different substrates. For kinetic assays, the protein was preserved in 50 mM Tris buffer (50 mM Tris, 10% glycerol). The protein concentration was measured using a Pierce1 BCA protein assay kit (Thermo Fisher Scientific) and recorded on an Enspire Multimode Plate Reader (PerkinElmer, MA, USA).

### In vitro assays and kinetic measurements

For measurement of terpene synthase activity, reactions were carried out using 10 μM AaTPS, 100 μM substrates (GPP, FPP, or GGPP), and 2 mM Mg^2+^ in 200 μL 50 mM Tris-HCl buffer (pH 7.6) with 10% glycerol at 30 °C overnight. The products were extracted with an equal volume of hexane and then detected and analyzed by GC/MS. To evaluate the effects of temperature and pH value on the activity of AaTPS, the temperature was varied between 20–42 °C and the pH was varied between 5.6 and 10.6. The concentration of AaTPS was held constant at 5 μM. For steady-state kinetics, terpene synthase activity was determined by the consumption of NADPH and the production of PP_i_. The reactions were performed in 96-well microplates and monitored by the absorbance at 340 nm. The reactions contain 50 μL of pyrophosphate reagent (Sigma, P7275) and 40 μL of buffer (50 mM Tris-HCl, 5 mM MgCl_2_, pH 7.6 and different concentrations of enzyme). The reaction mixtures were pre-incubated at 30 °C for 5 min. Then, the enzyme reactions were started by adding 10 μL of different concentration of FPP. Released PP_i_ was detected by using pyrophosphate reagent, which conducts cascade enzyme reactions of PP_i_-dependent fructose-6-phosphate, aldolase, triosephosphate isomerase, and α-glycerophosphate dehydrogenase. The activity was measured as the difference in the decrease in absorbance between the samples and the blanks per minutes. The extinction coefficient (ɛ_340_) for NADPH is 6.22 × 10^3^ M^−1^ mL^−1^. The definition of one unit of activity is the required amount of enzyme to consume 2 μM of NADPH and release 1 μM of PP_i_. Prenyltransferase activity was determined by the consumption of indole during the enzyme reaction. The reactions were monitored at 280 nm with HPLC. Reaction mixtures were prepared in a 1.5 mL tube with 45 μL of reaction volume (50 mM Tris-HCl (pH8.6), 5 mM MgCl_2_, 200 μM DMAPP and 20 μM enzyme). Reaction mixtures were preheated at 30 °C for 5 min. Next, 5 μL of indole (10x various concentrations) was added to each mixture to start the reaction. Reaction mixtures without enzyme were regarded as negative controls. Curve fitting was performed by using Prism 7 for Mac OS X, GraphPad Software, Inc.

To evaluate the promiscuity of AaTPS, the in vitro reaction was carried out using 50 μM AaTPS, 0.2 mM substrates (DMAPP, GPP or FPP), 1 mM indole or its analogues (tryptophan, 3-methylindole and 6-methylindole), 2 mM Mg^2+^, and 5 μL DMSO in 200 μL of 50 mM PB buffer (pH 7.6) at 28 °C overnight. To test the universal feature of indole prenyltransferase activity, in vitro assay was carried out using 50 μM terpene synthase (AaTPS, FgFS, FgJ01056, FgJ09920, FgGS, FgMS, CgDS and TXS), 200 μM substrates (DMAPP or GPP), 1 mM indole, 2 mM Mg^2+^, and 5 μL DMSO in 200 μL 50 mM PB buffer (pH 7.6) at 28 °C overnight. The products were extracted with an equal volume of hexane and then detected and analyzed by GC/MS. All reactions were performed in triplicate.

### Protein purification for AaTPS crystallization

Harvested cells were resuspended in 20 mL of lysis buffer [50 mM Tris-HCl (pH 7.6), 300 mM NaCl, 10% glycerol, 20 mM imidazole] with additional 1 mg mL^−1^ lysozyme, and sonicated for 30 min using a 50% duty cycle and power range of 30%. After 30 cycles of sonication, cell lysate was clarified by centrifugation at 15,000*g* at 4 °C for 30 min. The clarified lysate was loaded on a 5 mL of pre-equilibrated Ni-NTA resin with lysis buffer (4 °C). The resin was washed with 50-column volume of lysis buffer, and the proteins were eluted with 5 column volume of lysis buffer containing 300 mM imidazole. The pooled fraction containing the desired protein was concentrated and applied to HiLoad 16/60 Superdex 200 prepacked gel filtration column (4 °C, GE Healthcare), eluted with 20 mM Tris-HCl (pH 7.6), 100 mM NaCl, 1 mM dithiothreitol and 2 mM MgCl_2_, and concentrated to 19 mg ml^−1^ using Amicon Ultra-4 (MWCO: 30 kDa, Millipore) at 4 °C. The protein concentration was calculated by measuring UV absorption at 280 nm and using the extinction coefficients of AaTPS (47120 M^−1^ cm^−1^).

### Crystallization and structure determination of AaTPS

Well-diffracting AaTPS crystals were obtained at 10 °C, in 100 mM HEPES (pH 7.5), 2.0 M Ammonium formate (solution A), and 15–19 mg ml^−1^ of purified AaTPS through the vapor diffusion method. AaTPS crystals were soaked in solution A containing 1 mM FPP for 60 min at 20 °C. The crystals were transferred into the reservoir solution with 25% (v/v) Polyethylene glycol 400, and then flash cooled at −173 °C in a nitrogen-gas stream. X-ray diffraction data sets were collected at the BL-1A (Photon Factory, Tsukuba, Japan) and TPS-05A (NSRRC, Taiwan). We used wavelength of 1.7711 Å for Native-SAD method, and 0.9800 or 1.0000 Å was used for data collection of apo and PPi complex structures.

Diffraction data for Native-SAD method were processed and scaled using the XDS program package^43^. S sites were determined with AutoSol in PHENIX^44,45^. The sites were refined and the initial phases were calculated with AutoBuild in PHENIX^45,46^. The initial phases of the AaTPS binary complex structure were determined by molecular replacement, using the apo structure of AaTPS as a search model. Molecular replacement was performed with Phaser in PHENIX^45,47^. The structure was modified manually with Coot^48^ and refined with PHENIX^45,49^. The final crystal data and intensity statistics are summarized in Supplementary Dataset 2. The AaTPS binary complex structure contains PP_i_ in the active site, suggesting that FPP was hydrolyzed during the soaking. The Ramachandran statistics are as followed: 97.4% favored, 2.6% allowed, and 0% outliers for AaTPS apo; 96.3% favored, 3.6% allowed, and 0.1% outliers for AaTPS complexed with PP_i_. The B factors of AaTPS are higher than 50 Å^2^ and could due to the flexibility and low quality data. A structural similarity search was performed, using the Dali program^50^. All crystallographic figures were prepared with PyMOL (DeLano Scientific, http://www.pymol.org).

### Expression and purification of FgGS

A codon optimized gene encoding full-length FgGS (residues 1–316) was amplified using Pfu Ultra II Fusion polymerase (forward and reverse primers listed in Supplementary Table 5) and cloned into a pHIS parallel expression vector using the 5′ BamHI and 3′ XhoI sites and the Rapid DNA Ligation Kit (ThermoFisher)^51^. The gene was inserted in frame with a Tobacco Etch Virus (TEV) protease-cleavable N-terminal 6xHis tag. The cloned gene was transformed into NEB 5-alpha competent *E. coli* cells (New England Biolabs) and sequencing was used to confirm the correct insertion and orientation of the gene. The gene was transformed into BL21 (DE3) competent *E. coli* cells to express the protein. Cells were stored at −80 °C in 25% glycerol.

From the glycerol stock, plasmid-containing cells were grown in 70 mL of 2x YT medium supplemented with 50 mg L^−1^ carbenicillin, and the culture was allowed to grow overnight at 37 °C with shaking at 250 r.p.m. A 5 mL sample of the saturated culture was added to 1 L of 2x YT supplemented with 50 mg L^−1^ carbenicillin. The 1 L cell culture was allowed to grow at 37 °C with shaking at 250 r.p.m. When OD_600_ = 0.8, the culture was removed from the shaker and allowed to remain at 4 °C for 1 h. The flask was returned to the shaker and induced with 250 μM MgCl_2_ (Fisher Scientific) and 500 μM isopropyl-β-D-1-thiogalactopyranoside (IPTG; GoldBio) and allowed to incubate at 16 °C with shaking at 250 rpm for 16–18 h. Cells were pelleted by centrifugation at 5422*g* and stored at −80 °C.

Prior to purification, cell pellets were thawed at room temperature for 45 min. Lysis buffer [50 mM 4-(2-hydroxyethyl)-1-piperazineethanesulfonic acid (HEPES) (pH 7.75), 250 mM NaCl, 10% glycerol, 1.5 mM tris(2-carboxyethyl)phosphine (TCEP), 10 μM MgCl_2_, 30 mM imidazole, 0.3 mg mL^−1^ lysozyme (MPbiomedicals), 0.1 mg mL^−1^ DNase (Roche Applied Science), and protease inhibitor tablets (Roche Applied Science)] was added in a 2:1 ratio of buffer to pellet (v/w) and allowed to stir at 4 °C for 1 h. Cells were lysed by sonication and clarified by centrifugation at 38,000*g* at 4 °C for 1 h. Supernatant was loaded onto a 5-mL HisTrap (GE) column pre-equilibrated with loading buffer [50 mM HEPES (pH 7.75), 250 mM NaCl, 10% glycerol, 1.5 mM TCEP, 10 µM MgCl_2_, and 30 mM imidazole] and eluted with elution buffer [50 mM HEPES (pH 7.75), 250 mM NaCl, 10% glycerol, 1.5 mM TCEP, 10 µM MgCl_2_, and 500 mM imidazole] over a 25-column volume gradient. Appropriate fractions under the A_280_ peak were pooled and 7 mg of TEV protease was added and allowed to digest overnight while dialyzing into loading buffer. The protein digest was then loaded onto a HisTrap column pre-equilibrated with loading buffer and the flow-through was collected. The flow-through was concentrated and then loaded onto a HiLoad 26/600 Superdex 200 size-exclusion column (GE Healthcare) pre-equilibrated with size-exclusion buffer [50 mM HEPES (pH 7.5), 250 mM NaCl, 5% glycerol, and 1.5 mM TCEP]. Appropriate fractions were analyzed for purity using SDS-PAGE, pooled, concentrated, flash cooled, and stored at −80 °C.

### Crystallization and structure determination of FgGS

Crystals of the FgGS–Mg^2+^_3_–PP_i_–benzyltriethylammonium cation (BTAC) complex were grown by the sitting drop vapor diffusion at 4 °C. Utilizing a Mosquito crystallization robot (TTP Labtech), a 100 nL drop of 10 mg mL^−1^ FgGS in size-exclusion buffer supplemented with 2 mM BTAC (as the chloride salt), 10 mM MgCl_2_, and 2 mM sodium pyrophosphate was added to a 100 nL drop of precipitant solution [0.2 M NaCl, 0.1 M Bis–Tris (pH 5.5), and 25% w/v PEG 3350] and equilibrated against 80-µL of precipitant solution in the well reservoir. Crystals of the FgGS–Mg^2+^_3_–PP_i_–imidazolium complex were grown in similar fashion: a 1-µL drop of 5 mg mL^−1^ FgGS in size-exclusion buffer supplemented with 2 mM indole, 10 mM MgCl_2_, 2 mM sodium pyrophosphate, and 100 mM (NH_4_)_2_SO_4_ was added to a 1-µL drop of precipitant solution [0.1 M imidazole (pH 7.3), 24% PEG-monomethyl ether 5K, and 2% PEG 400] and equilibrated against 500-µL of precipitant solution in the well reservoir.

Both crystals were briefly immersed in a cryoprotectant solution containing their respective mother liquors supplemented with 15% ethylene glycol (v/v) before being flash cooled.

X-ray diffraction data from crystals of the FgGS–Mg^2+^_3_–PP_i_–BTAC complex were collected on Northeastern Collaborative Access Team (NE-CAT) beamline 24-ID-E at the Advanced Photon Source, Argonne National Laboratory (Argonne, IL). Data were integrated and scaled using iMosflm and Aimless in the CCP4 suite^52–54^. The initial electron density map was phased using molecular replacement with the cyclization domain of fusicoccadiene synthase (PDB 5ERT)^29^ less ligands and water molecules as a search probe using routines implemented in Phaser^47^. Following phasing, a RaptorX model of FgGS was aligned on the search probe^55^. Iterative model building and refinement of the protein model utilized COOT and Phenix, respectively^45,56^. The quality of the final model was assessed using MolProbity^57^.

X-ray diffraction data from crystals of the FgGS–Mg^2+^_3_–PP_i_–imidazole complex were collected on beamline 9–2 at the Stanford Synchrotron Radiation Lightsource (SSRL), SLAC National Accelerator Laboratory (Menlo Park, CA). Diffraction data were processed as described above, and the initial electron density map was phased by molecular replacement using the protein structure in the FgGS–Mg^2+^_3_–PP_i_–BTAC complex as a search probe with Phaser^47^. The structure was refined in similar manner as described above.

Segments from the N- and C-termini of monomer A (M1–Y4 and L309–G316, respectively) as well as segments from the N- and C-termini of monomer B (M1–S5 and D304–G316, respectively) in the FgGS–Mg^2+^_3_–PP_i_–BTAC complex were characterized by very weak or missing electron density and were excluded from the final model. Similarly, electron density was weak or missing from segments in the N- and C-termini of monomer A (M1–Y4 and L309–G316, respectively) as well as monomer B (M1–P3 and Y305–G316, respectively) in the FgGS–Mg^2+^_3_–PP_i_–imidazole complex. A loop in monomer B lacked electron density for the T102–V108 segment in the FgGS–Mg^2+^_3_–PP_i_–BTAC complex. The same loop in monomer B in the FgGS–Mg^2+^_3_–PP_i_–imidazole complex was missing electron density for residues D101–Q109. Although a segment of the C-terminus is missing in the FgGS–Mg^2+^_3_–PP_i_–BTAC complex, a disulfide bond appears to form between C60 and C312 in monomer B due to a strong peak adjacent to C60 in the electron density map. Regardless, we were unable to model the corresponding C-terminal segment which remains largely disordered. Extraneous electron density that was not interpretable was left unmodeled. Refined structures were validated with MolProbity^57^. Final refinement statistics are recorded in Supplementary Dataset 2. The Ramachandran statistics are as followed: 98.8% favored, 1.2% allowed, and 0% outliers for the FgGS–Mg^2+^_3_–PP_i_–BTAC complex; 98.6% favored, 1.4% allowed, and 0% outliers for the FgGS–Mg^2+^_3_–PP_i_–imidazole complex.

### Site-directed mutagenesis

Site-directed mutagenesis of AaTPS was performed using a QuikChange Site-Directed Mutagenesis Kit (Stratagene). The used primers were listed in Supplementary Table 5. The pET28a plasmid with AaTPS insert were used as templates for the mutagenesis reactions. The resulting plasmids were transformed into *E. coli* BL21 (DE3). All mutants were expressed and purified following the method described above.

### Enzymatic reaction assay for GC-MS analysis

The enzymatic reactions of AaTPS wild type and mutants with FPP were performed in a total volume of 50 μL containing 50 μM enzyme, 100 μM FPP, 50 mM Tris-HCl buffer (pH 7.1), and 2 mM MgCl_2_ for 2 h at 30 °C. The enzymatic reactions catalyzed by wild-type and mutant AaTPS enzymes using co-substrates indole and DMAPP were performed in a total volume of 50 μL containing 50 μM enzyme, 100 μM indole (in DMSO) and DMAPP, 50 mM Tris-HCl buffer (pH 8.5), 2 mM MgCl_2_ at 30 °C for 2 h. The reaction mixtures were analyzed by GC-MS.

### In vitro assays for other aromatic substrates

The enzymatic reactions catalyzed by AaTPS using aromatic substrates **15**–**25**, along with co-substrate DMAPP, were performed in a total volume of 100 μL containing 80 μM enzyme, 100 μM substrates (in DMSO), 100 μM DMAPP, 50 mM Tris-HCl buffer (pH 8.5), 4 mM MgCl_2_ at 30 °C overnight. The other 50 μL of the reaction mixtures were mixed with equal volume of CH_3_CN, then analyzed with LC/MS. The column used was a X-select CSH C_18_ 35 μm, 21 × 150 mm. The solvent was CH_3_CN-H_2_O (0.1% formic acid). Time program: 0–15 min, 30–100%;15–20 min, 100%; 20–21 min, 100–30%; 21–25 min, 30%.

### Chemical synthesis of compound 11 and 14

Compounds **11** and **14** were chemical synthesized by using indole and GPP as reactants. Under a N_2_ atmosphere, indole (0.20 mmol) was dissolved in THF (1.0 mL). After cooling to 0 °C, NaH (1.5 equiv) was added, and the mixture was stirred at this temperature for 40 min. Prenyl bromide or geranyl bromide (2 equiv) was then added dropwise at 0 °C, and the mixture was stirred at room temperature for 3 h. The reaction was quenched by water and extracted with dichloromethane. The organic phase was dried over sodium sulfate and concentrated under reduced pressure. Purification by semi-preparative HPLC yielded the desired product.

### Reporting summary

Further information on research design is available in the Nature Research Reporting Summary linked to this article.

## Supplementary information

Supplementary Information

Descriptions of Additional Supplementary Files

Supplementary Data 1

Supplementary Data 2

Reporting Summary

## Data Availability

The data supporting the findings of this study are available within the article and its Supplementary Information Files or from the corresponding authors on reasonable request. The source data underlying Figs. 2d–e, and 3a–d and Supplementary Fig. 7 are provided as a Source Data file. Protein Data Bank (PDB): The coordinates and the structure factor amplitudes for the apo structure of AaTPS and FgGS, complexed with ligands were deposited under accession codes 6LCC^58^ [10.2210/pdb6LCC/pdb], 6LCD^59^ [10.2210/pdb6LCD/pdb], 6VYD^60^ [10.2210/pdb6VYD/pdb], 6W26^61^. [10.2210/pdb6W26/pdb]. Source data are provided with this paper.

## Source data

Source Data
