# Supplementary material for: Discovery of the cryptic function of terpene cyclases as aromatic prenyltransferases
Source: Nat Commun. 2020 Aug 7;11:3958. doi: 10.1038/s41467-020-17642-2 (PMC7414894; doi:10.1038/s41467-020-17642-2)
Supplement: Supplementary file 1 — Supplementary Information [file 41467_2020_17642_MOESM1_ESM.pdf]

# Supplementary Information

# Discovery of the cryptic function of terpene cyclases as aromatic prenyltransferases

Haibing He,[1,*] Guangkai Bian,[2,*] Corey J. Herbst-Gervasoni,[3,*] Takahiro Mori,[1] Stephen A. Shinsky,[3] Anwei Hou,[2] Xin Mu,[2] Minjian Huang,[2] Shu Cheng,[2] Zixin Deng,[2] David W. Christianson,[3,¶] Ikuro Abe,[1,¶] Tiangang Liu [2,4,¶]

[1] Graduate School of Pharmaceutical Sciences, University of Tokyo, Tokyo, Japan.

[2] Key Laboratory of Combinatorial Biosynthesis and Drug Discovery, Ministry of Education and School of Pharmaceutical Sciences, Wuhan University, Wuhan, China.

[3] Roy and Diana Vagelos Laboratories, Department of Chemistry, University of Pennsylvania, Philadelphia, PA 19104-6323, United States. [4] Hubei Engineering Laboratory for Synthetic Microbiology, Wuhan Institute of Biotechnology, Wuhan, China. * These authors contributed equally to this work.

¶ e-mail: chris@sas.upenn.edu, liutg@whu.edu.cn, abei@mol.f.u-tokyo.ac.jp.

**Table of Contents**

**Supplementary Tables 1-5.**

**Supplementary Figures 1-8.**

**Supplementary Notes (NMR data)**

**Supplementary References**

**Supplementary Tables**

**Supplementary Table 1**. The NMR data of compound **4**.

| Position | $^{13}C$ | $^{1}H$ | | | | |
|---|---|---|---|---|---|---|
| | δ(ppm) | δ(ppm) | Intensity | Multiplicity | HMBC correlation | COSY correlation |
| 1 | | | | | | |
| 2 | 124.57 | 6.84 | 1H | brs | 3, 9, 8 | |
| 3 | 114.50* | | | | | |
| 4 | 119.11 | 7.57 | 1H | dt (*J* = 7.9, 1.0 Hz) | 8, 6 | H-5 |
| 5 | 118.47 | 7.08 | 1H | ddd (*J* = 8.0, 6.9, 1.1 Hz) | 7, 9 | H-4, H-6 |
| 6 | 121.25 | 7.18 | 1H | ddd (*J* = 8.2, 6.9, 1.2 Hz) | 4, 8 | H-5, H-7 |
| 7 | 109.38 | 7.28 | 1H | m | 5, 9 | H-6 |
| 8 | 136.38 | | | | | |
| 9 | 128.09* | | | | | |
| 1’ | 43.95 | 4.64 | 2H | d (*J* = 7.0 Hz) | 2’, 2, 3’ | H-2’ |
| 2’ | 120.34 | 5.36 | 1H | t (*J* = 7.0 Hz) | | H-1’ |
| 3’ | 135.62 | | | | | |
| 4’ | 18.01 | 1.82 | 3H | s | 5’, 2’, 3’ | |
| 5’ | 25.65 | 1.75 | 3H | s | 4’, 2’, 3’ | |
| 1’’ | 24.08 | 3.44 | 2H | d (*J* = 6.8 Hz) | 3, 2’’, 2, 9, 3’’ | H-2’’ |
| 2’’ | 123.34 | 5.42 | 1H | t (*J* = 7.1 Hz) | | H-1’’ |
| 3’’ | 131.60* | | | | | |
| 4’’ | 17.79 | 1.77 | 3H | s | 5’’, 2’’, 3’’ | |
| 5’’ | 25.74 | 1.74 | 3H | s | 4’’, 2’’, 3’’ | |

*The singal was confirmed by HSQC and HMBC spectrum.

**Supplementary Table 2**. Steady-state kinetic parameters for FPP cyclization and indole prenylation* catalyzed by AaTPS WT and mutants

| substrate | | $K_M$/μM | $k_{cat}$/s$^{-1}$ | $k_{cat}/K_M$ (μM$^{-1}$s$^{-1}$) |
|---|---|---|---|---|
| FPP | AaTPS | 25.2 ± 2.5 | 3.6 ± 0.1 | 0.14 |
| | F146A | 70.9 ± 6.8 | 12.6 ± 0.4 | 0.18 |
| | F244A | 16.2 ± 1.3 | 2.2 ± 0.1 | 0.14 |
| | Y272A | 19.3 ± 2.1 | 2.2 ± 0.1 | 0.12 |
| indole | AaTPS | 125.3 ± 12.0 | (3.5 ± 0.1) x10$^{-3}$ | 2.8 x10$^{-5}$ |
| | F146A | 133.8 ± 12.7 | (5.1 ± 0.2) x10$^{-3}$ | 3.8 x10$^{-5}$ |
| | F244A | 107.8 ± 14.0 | (3.5 ± 0.2) x10$^{-3}$ | 3.3 x10$^{-5}$ |
| | Y272A | 145.6 ± 9.9 | (4.8 ± 0.1) x10$^{-3}$ | 3.3 x10$^{-5}$ |

* DMAPP was used as a co-substrate to measure the kinetics of indole prenylation.

**Supplementary Table 3**. The conversion rate of AaTPS WT and mutants with indole substrate

| | Conversion rate | Relative reaction efficiency |
|---|---|---|
| WT | 68 ± 0.6% | 1.00 |
| F146A | 86 ± 0.3% | 1.26 |
| F244A | 74 ± 0.3% | 1.09 |
| Y272A | 77 ± 1.3% | 1.13 |

**Supplementary Table 4**. Strains and plasmids used in this research.

| **Strains** | **Relevant genotype** | | | **Reference** |
|---|---|---|---|---|
| TPF6 | *Alternaria alternata* | | | [17] |
| BL21 (DE3) | *E. coli B F$^-$dcm ompT hsdSB(rB$^-$mB$^-$)gal* | | | Invitrogen |
| C1 | *E. coli* BL21:: pMH1, pFZ81 | | | [10] |
| GB171 | *A. alternata* TPF6:: *$p_{alcA}$-AaTPSgDNA* | | | This work |
| GB230 | *E. coli* BL21:: pMH1, pFZ81, pGB230 | | | This work |
| **Plasmids** | **Origin of replication** | **Description** | **Resistance** | **Reference** |
| pMH1 | p15A | plac: AtoB, ERG13, tHMG1 | CM | [10] |
| pFZ81 | pBBR1MCS | plac: ERG12, ERG8, MVD1, Idi | KAN | [10] |
| pGB308 | pBR322 | pT7: FPPS, Idi | AMP | [13] |
| pGB163 | pBR322 | pT7: N-terminal his$_6$-tag AaTPS | KAN | This work |
| pGB171 | pBR322 | pGB94; alcAp-AaTPS(gDNA) | AMP | This work |
| pGB230 | pBR322 | pT7: AaTPS, FPPS, Idi | AMP | This work |

**Supplementary Table 5.** Oligonucleotides used for construction of plasmids used in this research.

| Primer name | Primer Sequence (5'-3') |
|---|---|
| P1 | atatCATATGTCTCCTGACCTGACATACAC |
| P2 | atatGAATTCTATTTTTCCAGCTTGGCTGCCTTACC |
| P3 | atatTCTAGAGCCACCATGGGCAGCAGCCATC |
| P4 | atatGGTCACCCTATTTTTCCAGCTTGGCTGC |
| P5 | atatGGTGACCAGCTCGAATTTCCCCG |
| P6 | ctgcTCTAGATTATATAGATGTTCAGCTATGCGGGGAT G |
| P7 | atatCATATGAGCCCGGACCTGACCTATACC |
| P8 | atatGAATTCGGTCACCTTACTTTTCCAGTTTCGCCG |
| P9 | atatCTCGAGACTAGTTACTTTTCCAGTTTCGCCGCCTT G |
| AaTPS | Fw: CGCGCGGCAGCCATATGAGCCCGGACCTGAC<br>Rv: TGTCGAGTGCGGCCGCAAGCTTACTTTTCCAGTTTCG |
| *AaTPSF146A* | *Fw: GAGGAAGATCGTGCGACCAAATTTGCG;*<br>*Rv: CAAATTTGGTCGCACGATCTTCCTCGTCG* |
| *AaTPSF154A* | *Fw: CGACCTATATTGCGCCGGAAGCGAACG*<br>*Rc: GCTTCCGGCGCAATATAGGTCGCAAATTTGGTG* |
| *AaTPSY272A* | *Fw: GGCGTTCCCGGCGCTGCTGG*<br>*Rv: AGCGCCGGGAACGCCACATC* |
| *FgGS* | *Fw: AAACCATGGATATGGATCCCTACAGTG*<br>*Rv: GGGAAGCTTTTAACCCTCGTGCTGAC* |

**Supplementary Figures**

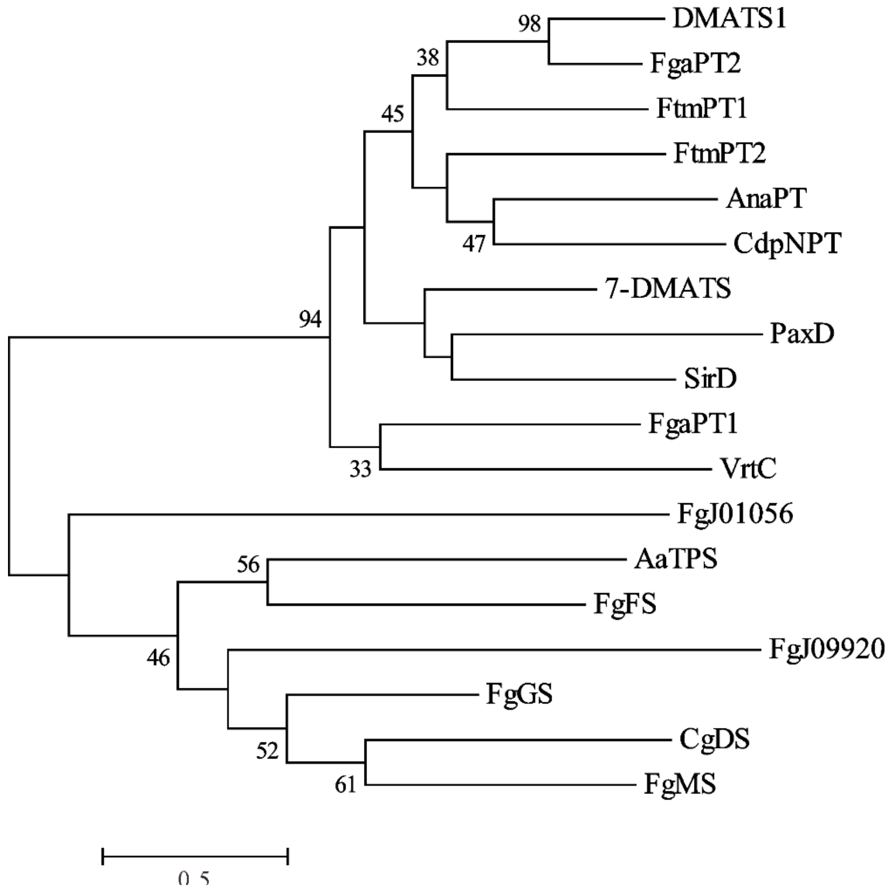


**Supplementary Figure 1**. Maximum likelihood method based phylogenetic analysis of terpene cyclases and soluble aromatic prenyltransferases in fungi. Terpene cyclase AaTPS from *Alternaria alternata*, FgMS, FgGS, FgJ09920, FgJ01056 and FgFS from *Fusarium graminearum*, CgDS from *Colletotrichum gloeosporioides*. Soluble aromatic prenyltransferase DMATS1 (Accession No. M1WA41) from *Claviceps purpurea*, AnaPT (A1DN10) from *Aspergillus fischeri*, PaxD (AAK11526) from *Penicillium paxilli*, VrtC (ADI24928) from *Penicillium aethiopicum*, SirD (AAS92554) from *Leptosphaeria maculans*, and FgaPT1 (XP_756136), FgaPT2 (AAX08549.1), FtmPT1 (AAX56314), FtmPT2 (ACF22981) , 7-DMATS (ABS89001) and cdpNPT (ABR14712) from *Aspergillus fumigatus*.

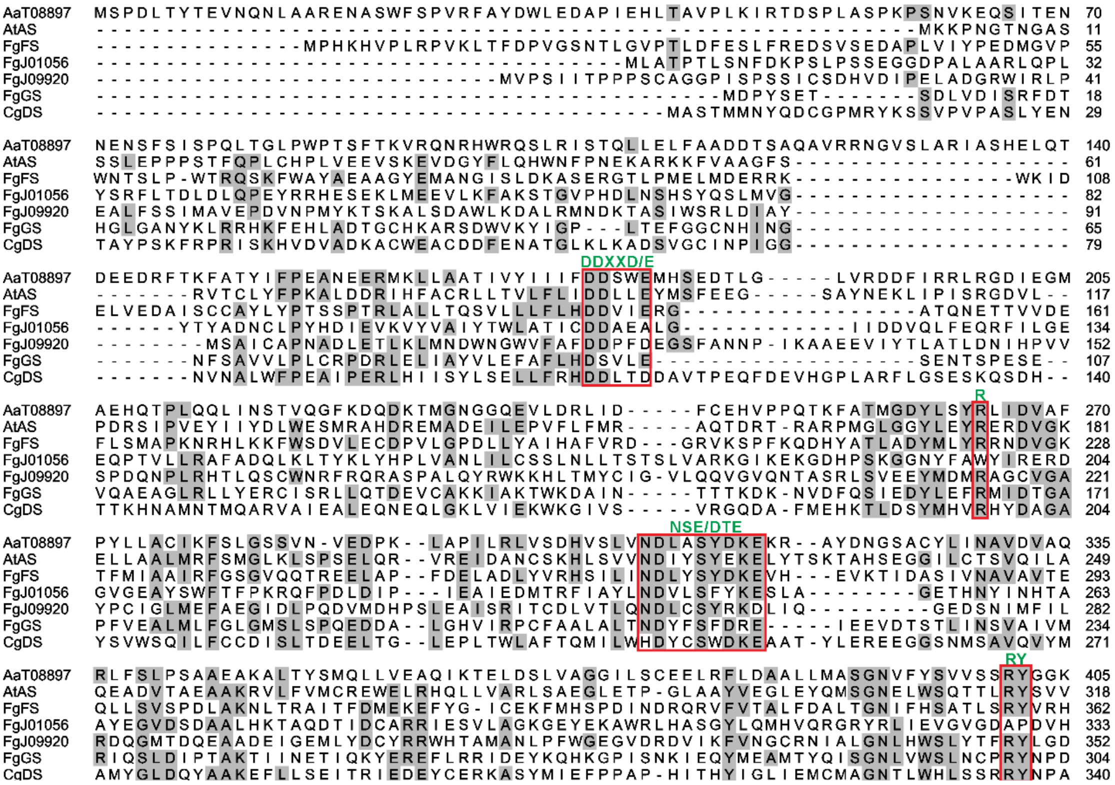


**Supplementary Figure 2**. Sequence alignment of AaTPS (AaT08897) with fungal class I terpene cyclases. The red boxes represent the conserved “DDXXD/E”, “NSE/DTE”, “R”, and “RY” motifs.

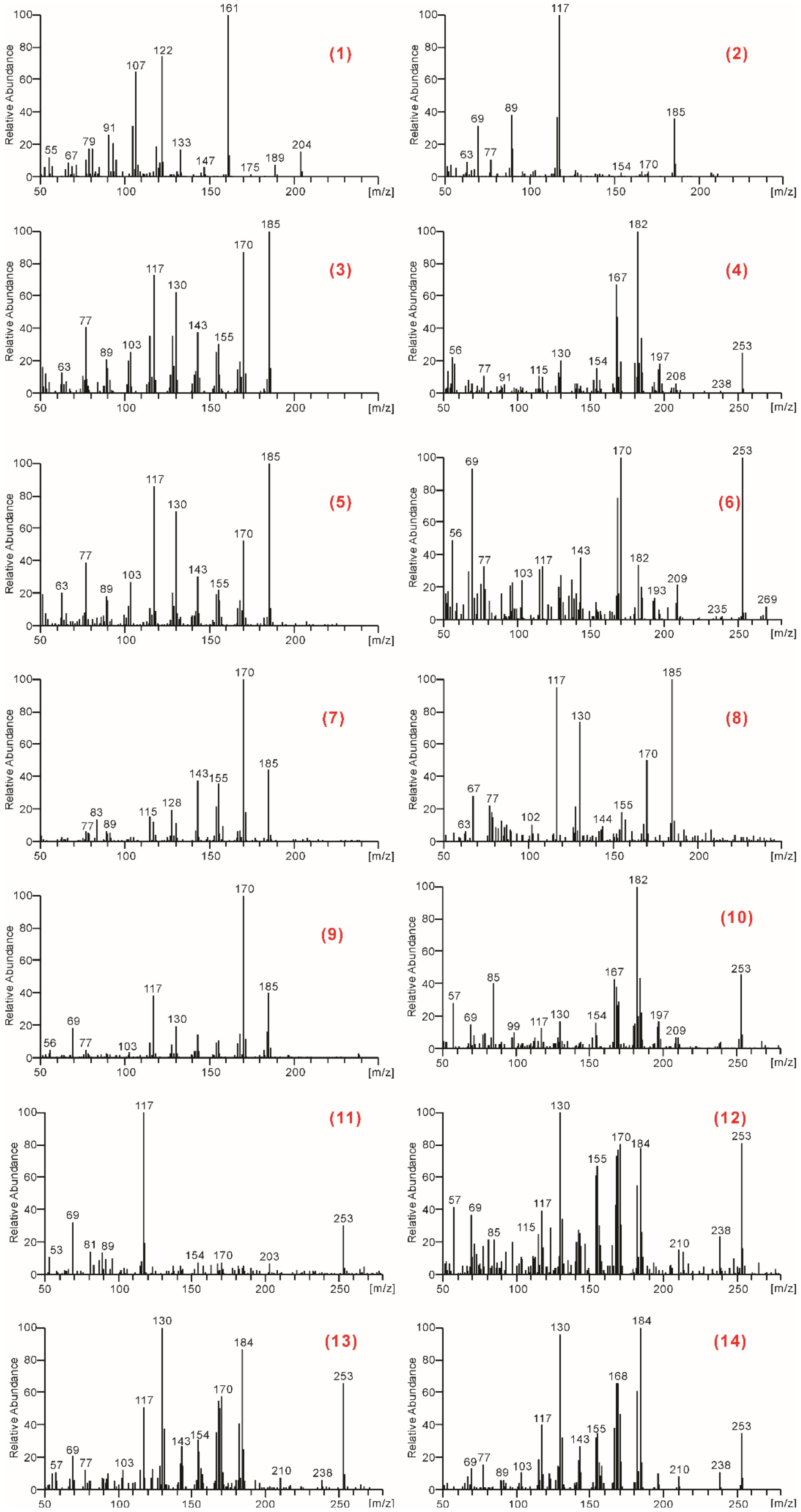


**Supplementary Figure 3**. Mass spectra of sesquiterpene and prenylindoles produced by in vitro assay of terpene synthases by using FPP, indole and DMAPP, or GPP as substrates.

a

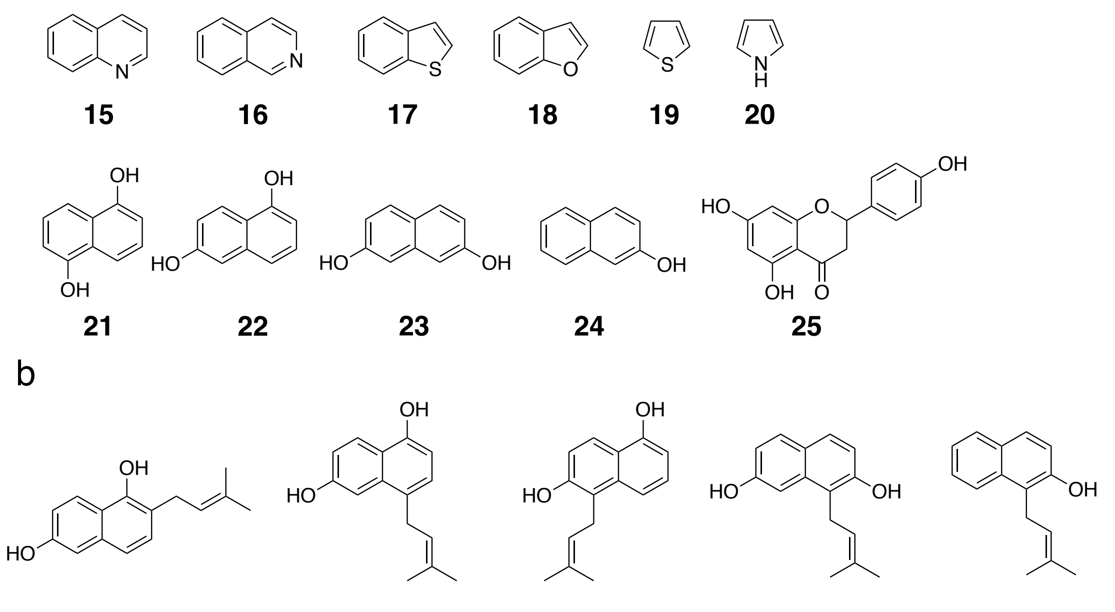

15 16 17 18 19 20

21 22 23 24 25

b

26 27 28 29 30

**Supplementary Figure 4**. a. Tested aromatic substrates. b. Aromatic products.

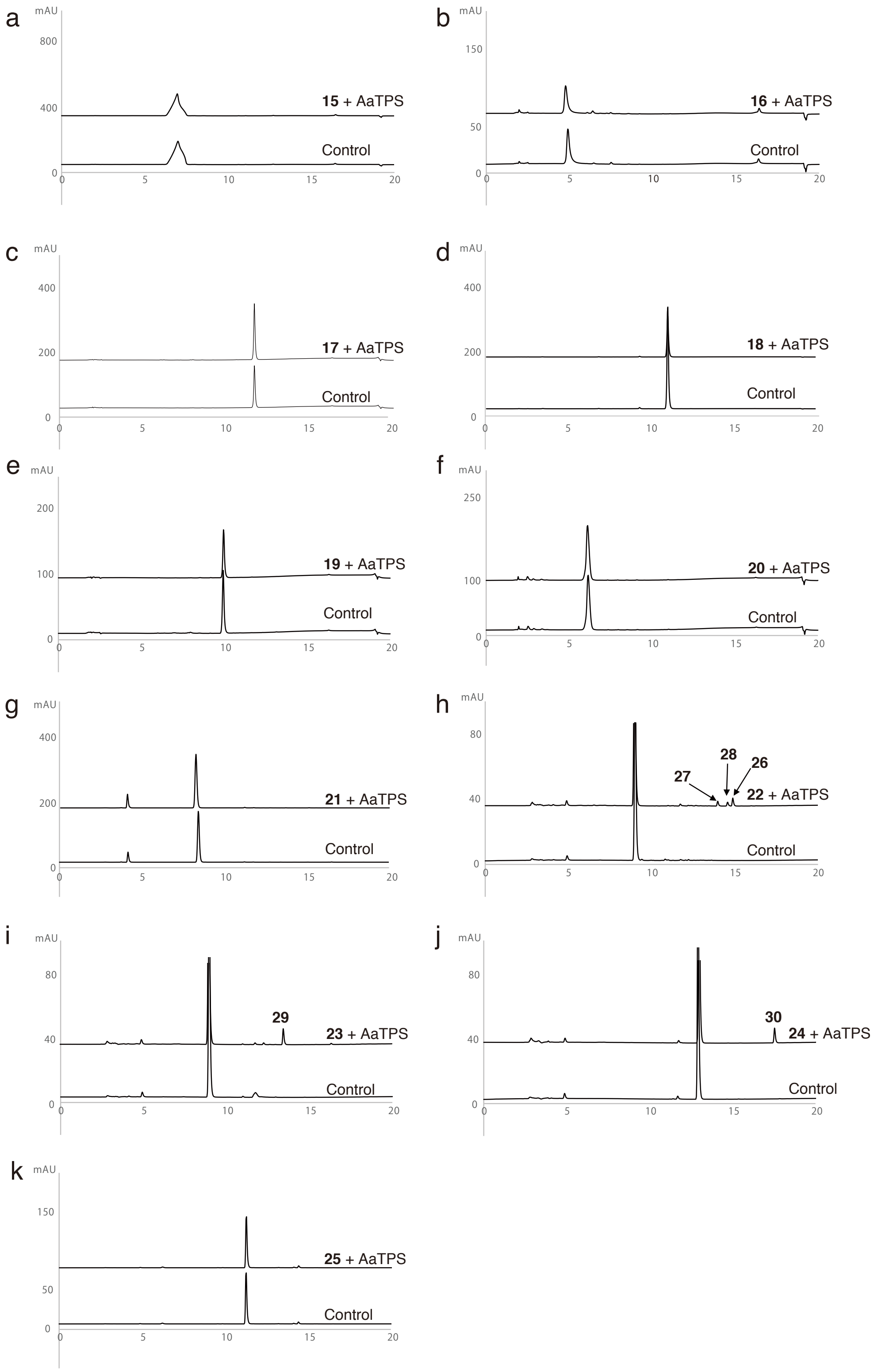


**Supplementary Figure 5.** HPLC chromatograms of the enzyme reactions of AaTPS with aromatic compounds. The structures of substrates and products are shown in Supplementary Figure 4.

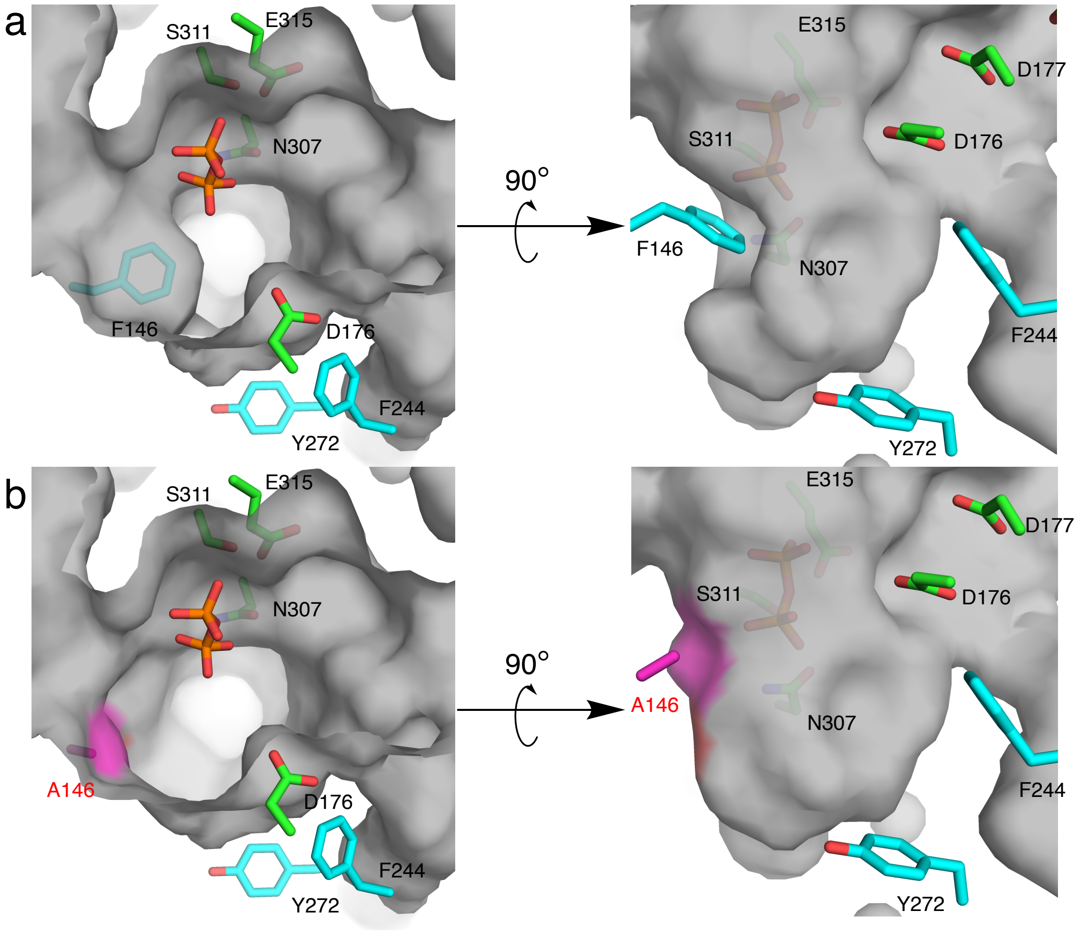


**Supplementary Figure 6.** (a) Molecular surface representation of the hydrophobic active site cavity of wild-type AaTPS, showing the positions of active site aromatic residues F146, F244, and Y272. (b) Molecular surface representation of the hydrophobic active site cavity in a model of F146A AaTPS. This mutant exhibits the largest change in turnover number ($k_{cat}$) among the mutants studied (three-fold increase for FPP cyclization); however, offset by an approximately three-fold increase in the Michaelis constant $K_M$, the catalytic efficiency ($k_{cat}/K_M$) of this mutant is comparable to that of the wild-type enzyme (**Supplementary Table 2**). The F146A substitution is closest to the binding site of the substrate diphosphate group, which may explain why this mutation shows the largest, yet nonetheless modest, effect on catalysis. The large-to-small substitution of F146 expands the hydrophobic cavity, resulting in slightly less contact in the precatalytic enzyme-substrate complex, which would be consistent with a three-fold increase in $K_M$. We speculate that less contact in this region of the active site could slightly enhance product release, which could account for the modest three-fold increase in $k_{cat}$ measured for F146A AaTPS. The DDXXD and NSE motifs were colored in green. In the figure above, mutated residues and modeled A146 are colored cyan and magenta, respectively.

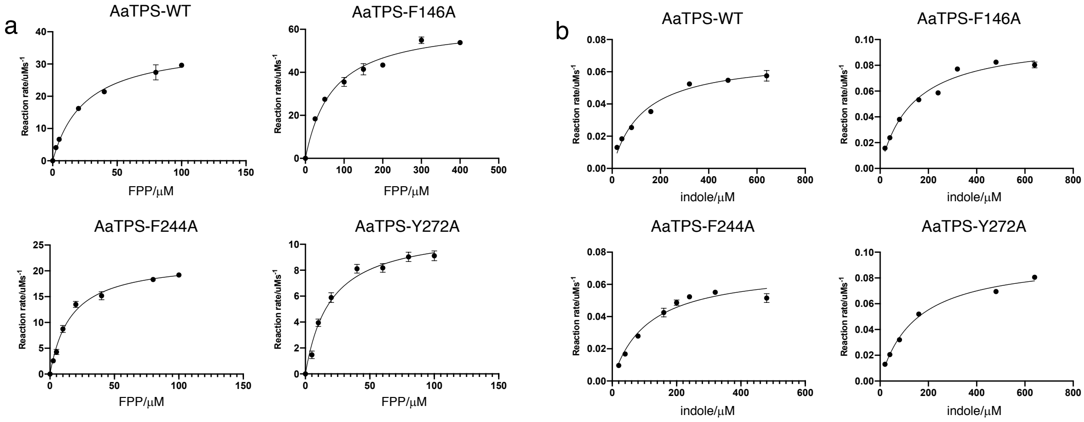


**Supplementary Figure 7.** Michaelis-Menten curves of AaTPS wild-type and its mutants for (a) FPP cyclization and (b) indole prenylation reactions. Error bars represent statistical differences (SDs) of three biological replicates.

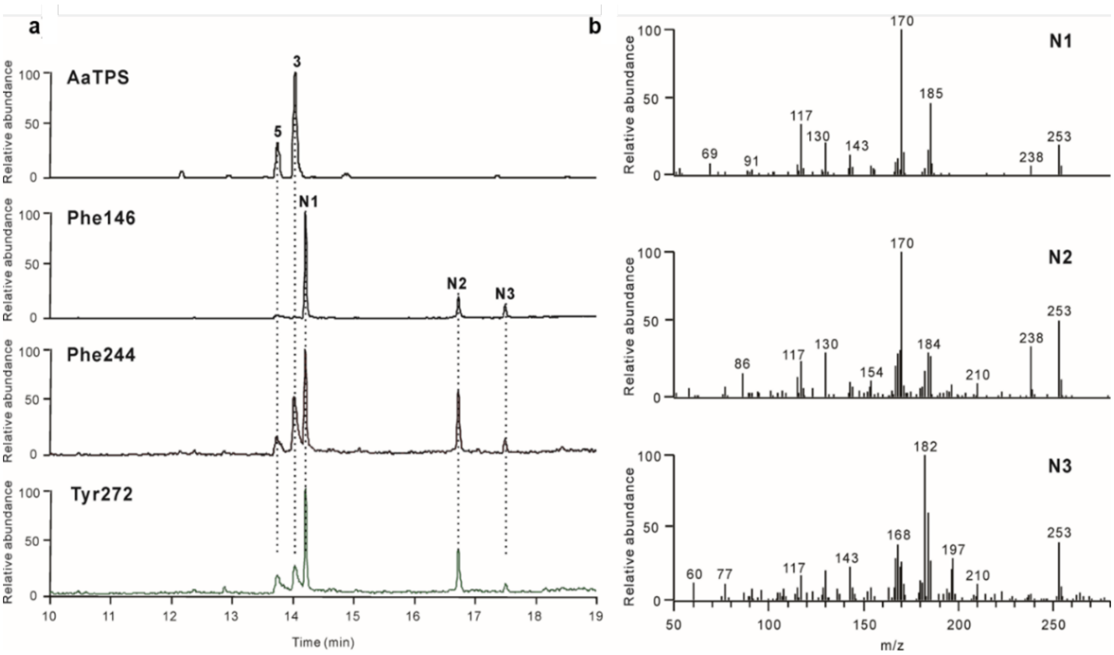


**Supplementary Figure 8.** GC-MS chromatograms of the enzyme reactions of AaTPS and mutants with indole compounds. a) represents the GC spectrum of compounds produced by AaTPS and mutants. Phe146, Phe244 and Tyr272 represent the mutant AaTPS-Phe146, AaTPS-Phe244 and AaTPS-Tyr272, respectively. N1, N2 and N3 represent the new prenylindoles produced by these three mutants. b) represents the mass spectrum of prenylindoles N1 to N3.

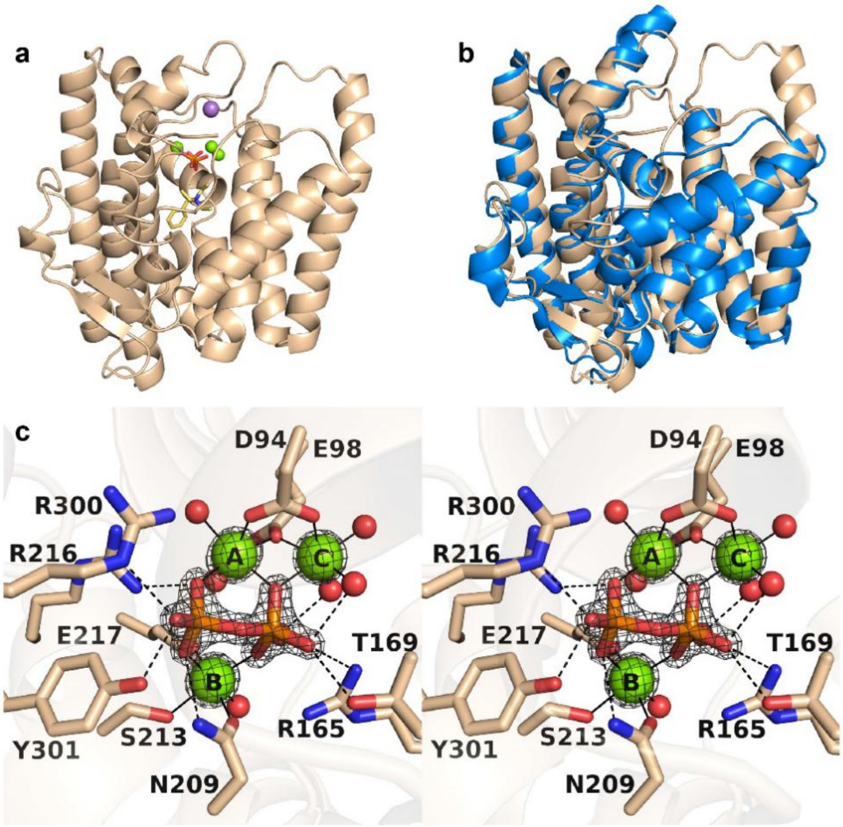


**Supplementary Figure 9.** (a) Ribbon plot of the FgGS-$Mg^{2+}{}_3$-$PP_i$-BTAC complex. The main chain is tan, $Mg^{2+}$ ions are green, and $PP_i$ and BTAC are color-coded by atom (C = yellow, N = blue, O = red, Na+ = purple, P = orange). (b) Superposition of the FgGS-$Mg^{2+}{}_3$-$PP_i$-BTAC complex (tan) with the fusicoccadiene synthase- $Mn^{2+}{}_3$-neridronate complex (PDB 5ER8; blue). Bound ligands are omitted for clarity. (c) Stereoview of Polder omit maps of the catalytic $Mg^{2+}$ ions (contoured at 9.0$\sigma$) and $PP_i$ (contoured at 16.0$\sigma$) in the FgGS active site. Metal coordination is indicated by solid black lines and hydrogen bonds are shown as dashed black lines.

**Supplementary Notes**

**NMR data for compounds 1-4, 11, 14, and 26-30.**

**Compound 1**: 7-epi-α-selinene[18]. $^1$H NMR (400 MHz, $CDCl_3$) δ 5.31 (dtd, *J* = 4.2, 2.8, 1.3 Hz, 1H), 4.90 (h, *J* = 1.6 Hz, 1H), 4.84 (td, *J* = 2.0, 0.9 Hz, 1H), 2.51 – 2.31 (m, 1H), 2.14 – 1.88 (m, 3H), 1.87 – 1.79 (m, 2H), 1.74 (dt, *J* = 1.5, 0.8 Hz, 3H), 1.60 (dq, J = 2.8, 1.5 Hz, 3H), 1.45 – 1.25 (m, 5H), 1.16 (dt, *J* = 13.1, 3.6 Hz, 1H), 0.84 (s, 3H). $^{13}$C NMR (101 MHz, $CDCl_3$) δ 147.01, 135.15, 121.16, 110.79, 41.18, 39.44, 38.24, 36.08, 32.83, 25.27, 23.39, 22.88, 22.84, 21.20, 15.52. HRESIMS (positive) *m/z* 205.1949 $[M{-}OH]^+$ (calcd. for $C_{15}H_{25}$, 205.1951).

**Compound 2**: 1-(3-methyl-2-butenyl)indole, yellow solid.$^1$H NMR (400 MHz, $CDCl_3$) δ = 7.71 (m, 1H), 7.57 (s, 2H), 7.39 (d, *J*=8.1, 0H), 7.24 (m, 1H), 6.98 (d, *J*=0.8, 0H), 5.54 (s, 0H), 3.55 (d, *J*=7.1, 1H), 1.87 (d, *J*=5.1, 3H). $^{13}$C NMR (101 MHz, $CDCl_3$) δ = 136.52, 132.01, 127.50, 123.16, 121.99, 121.28, 119.21, 119.10, 116.14, 111.16, 25.84, 24.18, 17.89. HRESIMS (positive) *m/z* 186.1275 $[M{-}OH]^+$ (calcd. for $C_{13}H_{16}N$, 186.1277).

**Compound 3**: 3-(3-methyl-2-butenyl)indole (Masanari Kimura et al, 2005), yellow solid.$^1$H NMR (400 MHz, $CDCl_3$) δ = 7.69 (d, *J*=7.9, 1H), 7.39 (d, *J*=8.2, 1H), 7.26 (dd, *J*=13.8, 6.0, 1H), 7.16 (q, *J*=6.0, 2H), 6.54 (d, *J*=3.1, 1H), 5.44 (m, 1H), 4.74 (d, *J*=6.9, 2H), 1.88 (s, 3H), 1.81 (s, 4H). $^{13}$C NMR (101 MHz, $CDCl_3$) δ = 136.23, 135.98, 128.74, 127.41, 121.32, 120.93, 120.03, 119.27, 109.53, 100.93, 44.18, 25.70, 18.07. HRESIMS (positive) *m/z* 186.1275 $[M{-}OH]^+$ (calcd. for $C_{13}H_{16}N$, 186.1277).

**Compound 4**: 1,3-bis(3-methyl-2-butenyl) indole.$^{1}$H NMR (400 MHz, $CDCl_3$) δ = 7.59 (d, *J*=7.9, 1H), 7.30 (d, *J*=8.2, 1H), 7.21 (t, *J*=7.2, 1H), 7.10 (t, *J*=7.1, 1H), 6.87 (s, 1H), 5.52 – 5.31 (m, 2H), 4.66 (d, *J*=6.8, 2H), 3.47 (d, *J*=7.0, 2H), 1.92 – 1.70 (m, 15H). $^{13}$C NMR (101 MHz, $CDCl_3$) δ = 136.44, 135.66, 131.59, 128.03, 124.61, 123.40, 121.30, 120.39, 119.16, 118.52, 114.56, 109.41, 43.99, 25.75, 25.66, 24.12, 18.03, 17.81. HRESIMS (positive) *m/z* 254.1901 $[M–OH]^+$ (calcd. for $C_{18}H_{24}N$, 254.1903).

**Compound 11**: $^{1}$H NMR (400 MHz, $CDCl_3$) δ = 7.67 (d, *J*=7.9, 0H), 7.38 (d, *J*=8.2, 0H), 7.24 (t, *J*=7.5, 0H), 7.14 (dd, *J*=7.9, 5.4, 1H), 6.52 (d, *J*=3.0, 0H), 5.43 (t, *J*=6.5, 0H), 5.10 (t, *J*=6.0, 0H), 4.74 (d, *J*=6.8, 1H), 2.13 (m, 1H), 1.85 (s, 1H), 1.71 (s, 1H), 1.62 (s, 1H). $^{13}$C NMR (101 MHz, $CDCl_3$) δ = 139.77, 136.01, 131.92, 128.74, 127.36, 123.77, 121.29, 120.87, 119.84, 119.24, 109.54, 100.85, 44.10, 39.47, 26.27, 25.71, 17.73, 16.39. HRESIMS (positive) *m/z* 254.1900 $[M–OH]^+$ (calcd. for $C_{18}H_{24}N$, 254.1903).

**Compound 14**: $^{1}$H NMR (400 MHz, $CDCl_3$) δ = 7.93 (s, 1H), 7.64 (t, *J*=8.0, 1H), 7.38 (d, *J*=8.1, 1H), 7.37 (t, *J*=8.2, 1H), 7.22 (t, *J*=7.1, 1H), 7.14 (t, *J*=7.5, 1H), 6.98 (s, 1H), 5.49 (t, *J*=7.1, 1H), 5.16 (t, *J*=6.7, 1H), 3.50 (d, *J*=7.1, 2H), 2.13 (m, 4H), 1.79 (s, 3H), 1.72 (s, 3H), 1.64 (s, 3H). $^{13}$C NMR (101 MHz, $CDCl_3$) δ = 136.51, 135.64, 131.42, 127.51, 124.40, 122.92, 121.92, 121.19, 119.14, 119.08, 116.18, 111.02, 39.72, 26.68, 25.75, 24.00, 17.73, 16.08. HRESIMS (positive) *m/z* 254.1900 $[M–OH]^+$ (calcd. for $C_{18}H_{24}N$, 254.1903).

**Compound 26** (SI ref. 1): $^1$H NMR (900 MHz, Methanol-d4) δ 8.04 (d, *J* =8.8 Hz, 1H, H-8), 7.00 (m, 2H, H-5, 7), 7.12 (d, *J* = 8.4 Hz, 1H, H-3), 7.13 (d, J = 8.4 Hz, 1H, H-4), 5.31 (m, 1H, H-2’), 3.43 – 3.39 (m, 2H, H-1’), 1.74 (d, *J* = 1.3 Hz, 3H), 1.72 (d, *J* = 1.4 Hz, 3H).

**Compound 27** (SI ref. 2): $^1$H NMR (500 MHz, Methanol-d4) δ 8.04 (d, *J* = 9.0 Hz, 1H, H-8), 7.15 (d, *J* = 2.4 Hz, 1H, H-5), 6.96 (dd, *J* = 9.0, 2.4 Hz, 1H, H-7), 6.49 (d, *J* = 7.6 Hz, 1H, H-2), 6.98 (d, *J* = 7.6 Hz, 1H, H-3), 5.34 – 5.27 (m, 1H), 3.64 – 3.60 (m, 2H), 1.76 (d, *J* = 1.3 Hz, 3H), 1.72 (d, *J* = 1.4 Hz, 3H).

**Compound 28** (SI ref. 3)**:** $^1$H NMR (500 MHz, Methanol-d4) δ 7.91 (d, *J* = 8.9 Hz, 1H, H-8), 6.98 (d, *J* = 9.0 Hz, 1H, H-7),7.28 (d, *J* = 8.6 Hz, 1H, H-4), 7.15 (dd, *J* = 8.5, 7.4 Hz, 1H, H-3), 6.57 (d, *J* = 7.4 Hz, 1H, H-2), 5.14 (m, 1H, H-2’), 3.66 (d, *J* = 6.8 Hz, 2H, H-1’), 1.84 (d, *J* = 1.3 Hz, 3H), 1.64 (d, *J* = 1.5 Hz, 3H).

**Compound 29** (SI ref. 3): $^1$H NMR (500 MHz, Methanol-d4) δ 7.55 (d, *J* = 8.8 Hz, 1H, H-4), 7.42 (d, *J* = 8.8 Hz, 1H, H-5), 7.09 (d, *J* = 2.2 Hz, 1H, H-8), 6.85 (d, *J* = 8.8 Hz, 1H, H-3), 6.81 (dd, *J* = 8.8, 2.2 Hz, 1H, H-6), 5.18 – 5.12 (m, 1H, H-2’), 3.63 – 3.59 (m, 2H, H-1’), 1.85 (d, *J* = 1.3 Hz, 3H), 1.65 (q, *J* = 1.5 Hz, 3H).

**Compound 30** (SI ref. 4): $^1$H NMR (500 MHz, Methanol-d4) δ 7.80 (d, *J* = 8.5 Hz, 1H,H-8), 7.68 (d, *J* = 8.3 Hz, 1H, H-5), 7.54 (d, *J* = 8.8 Hz, 1H, H-4), 7.36 (m, 1H, H-6), 7.21 (m, 1H, H7), 7.06 (d, *J* = 8.8 Hz, 1H, H-3), 5.16 (m, 1H, H-2’), 3.71 (d, *J* = 7.2 Hz, 2H, H-1’), 1.86 (s, 3H), 1.65 (s, 3H).

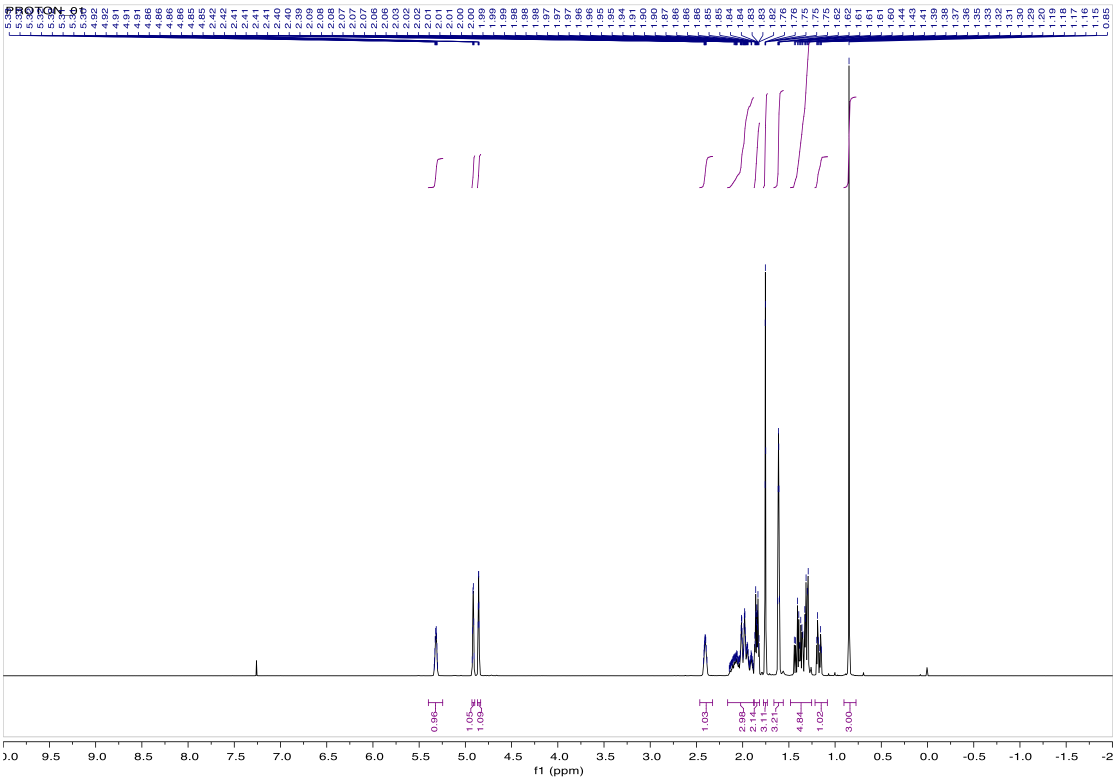

$^{1}H$ NMR of compound **1** (400Mz, $CDCl_3$).

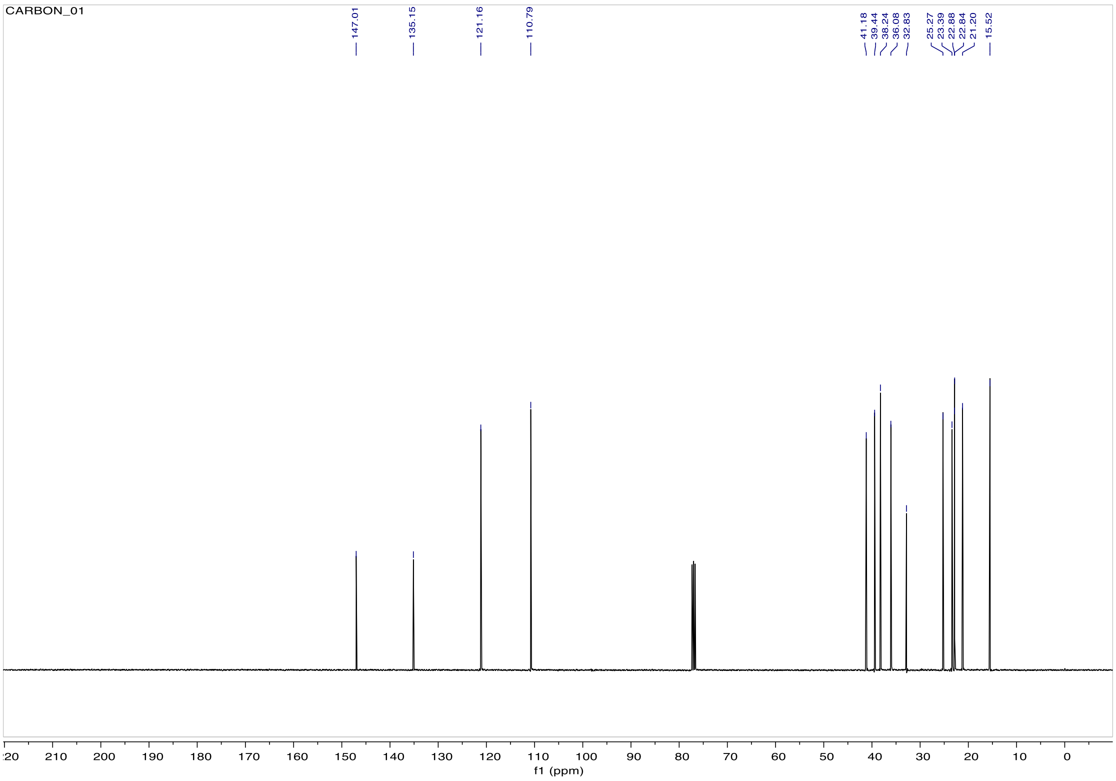

$^{13}C$ NMR of compound **1** (101Mz, $CDCl_3$).

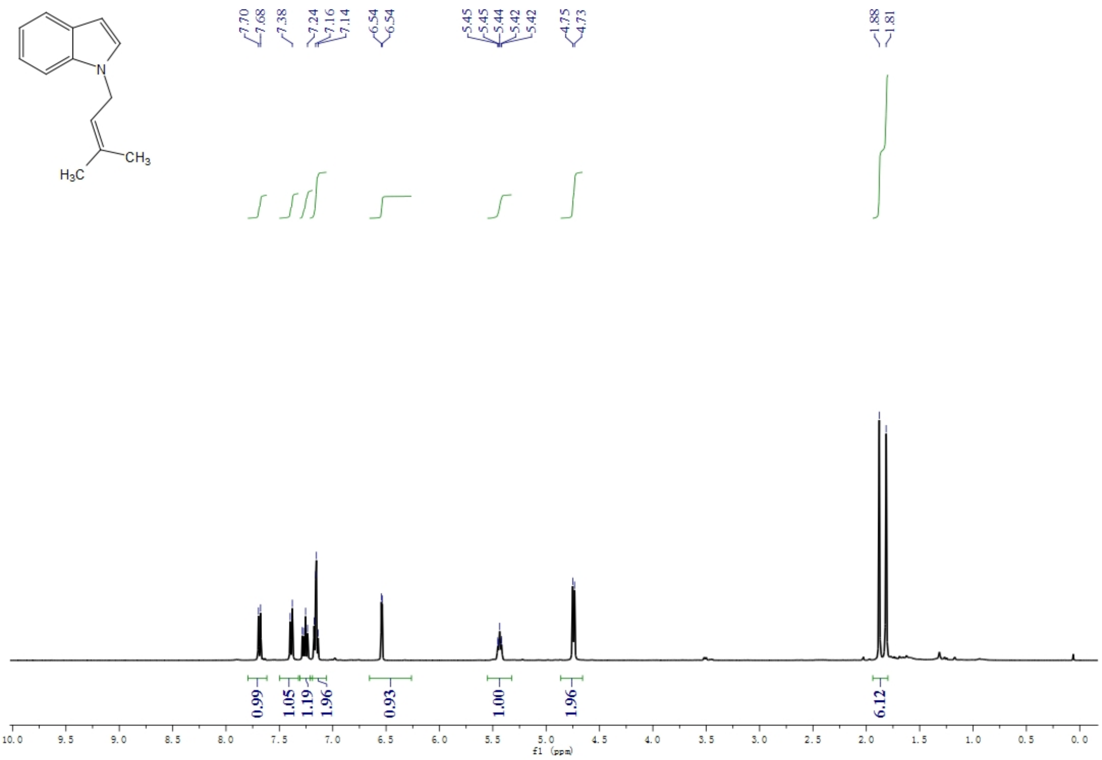


$^{1}$H-NMR spectrum of Compound **2**.

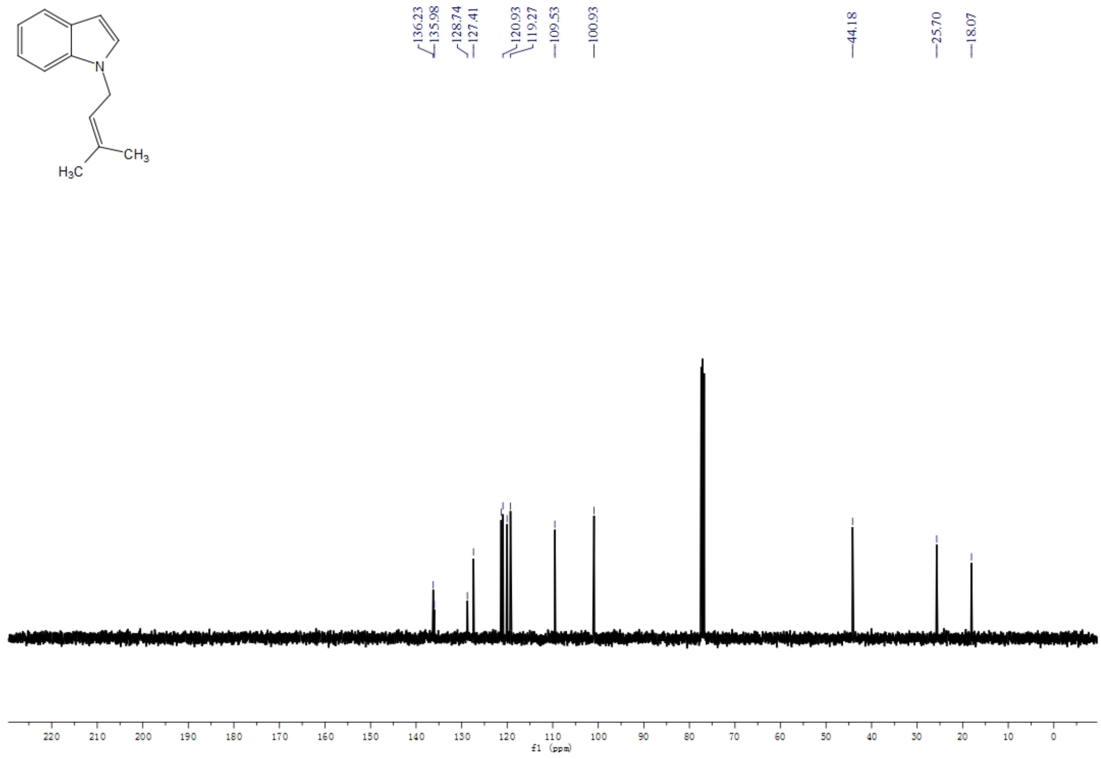


$^{13}$C-NMR spectrum of Compound **2**.

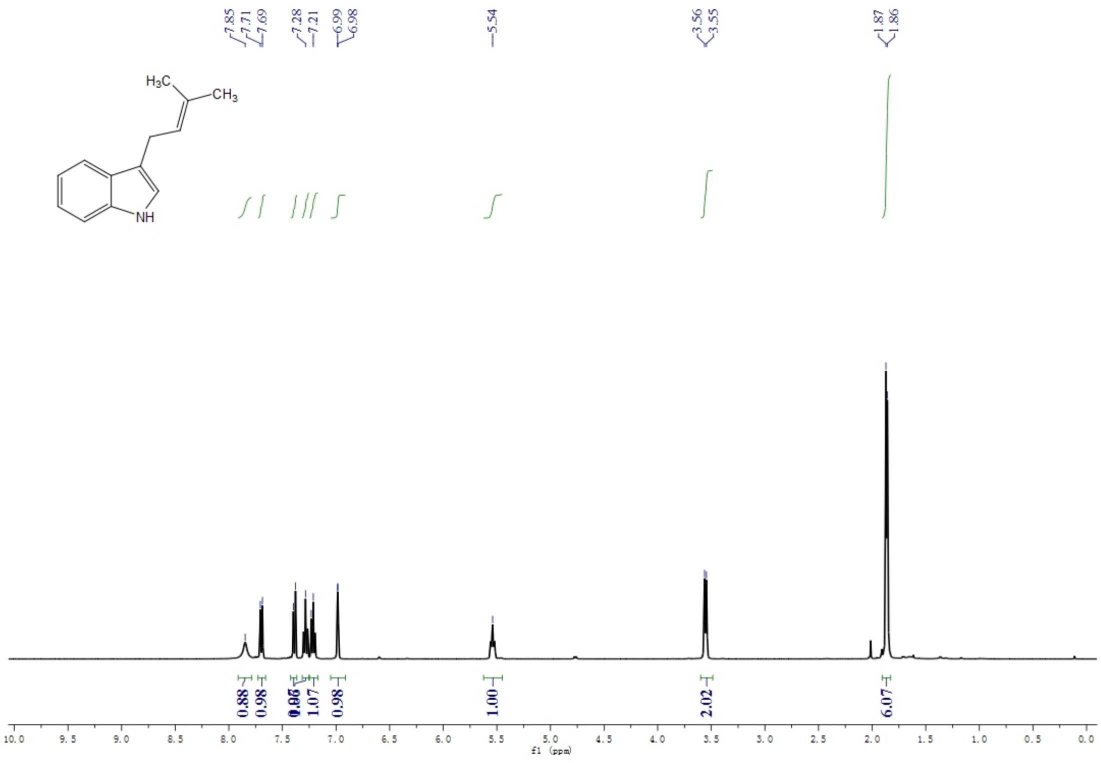

$^{1}$H-NMR spectrum of Compound **3**.

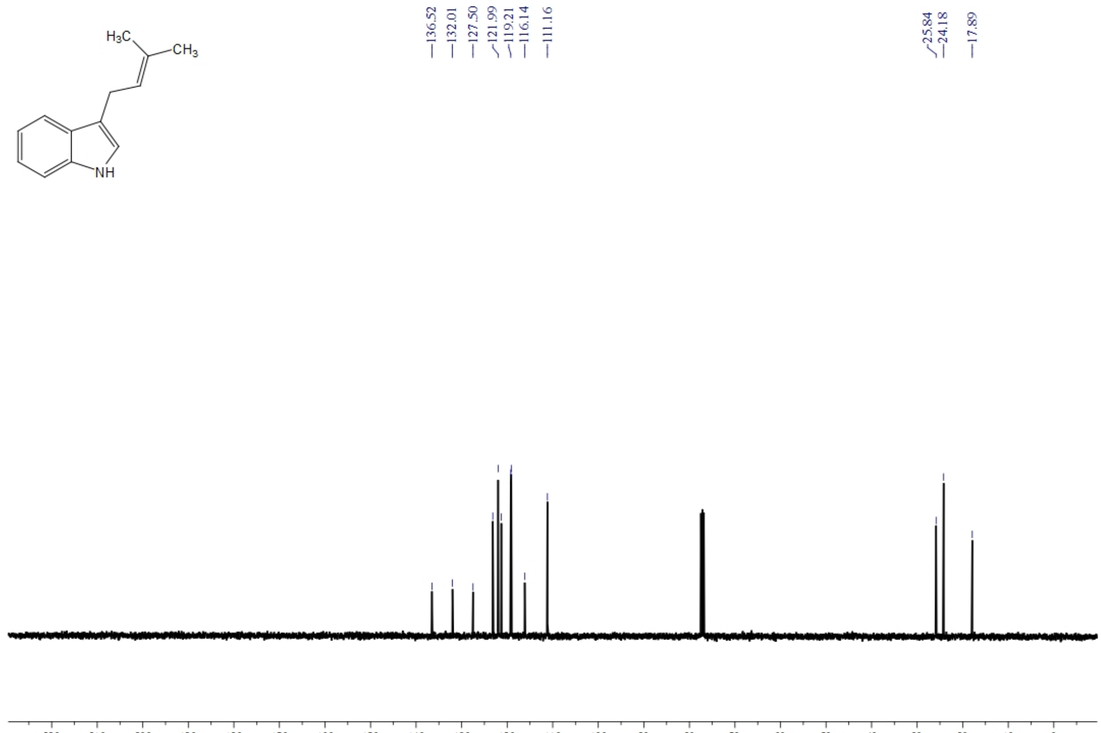

$^{13}$C-NMR spectrum of Compound **3**.

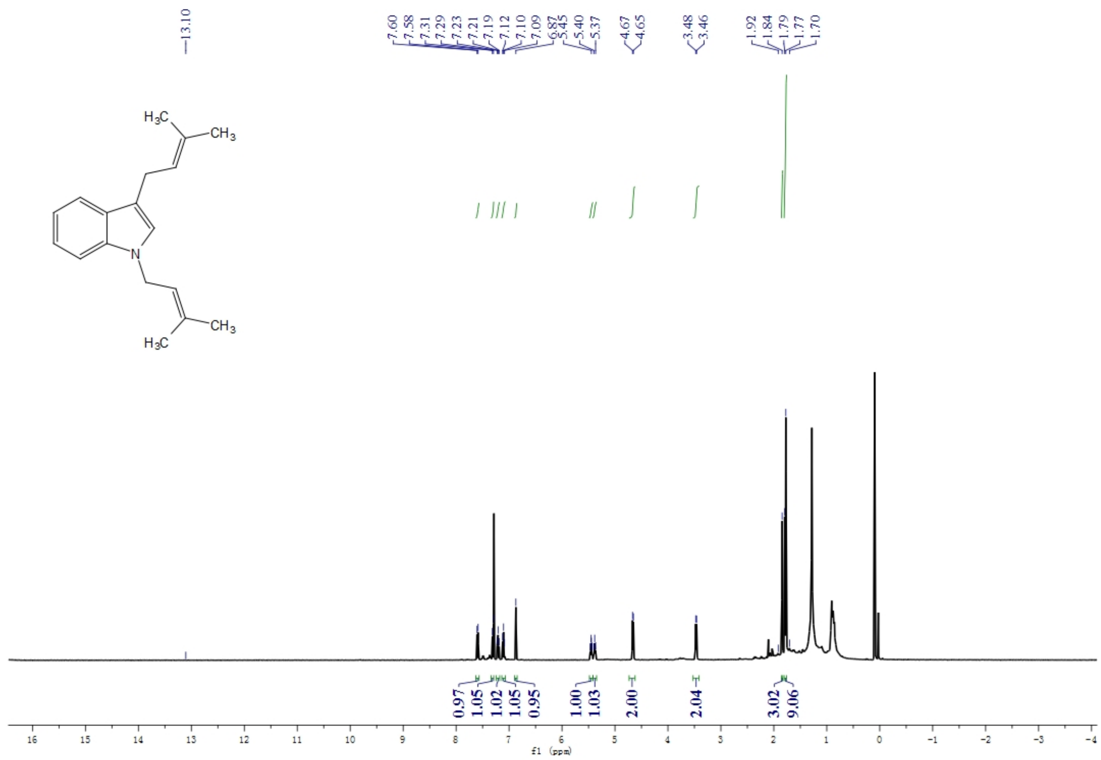


$^{1}$H-NMR spectrum of Compound **4**.

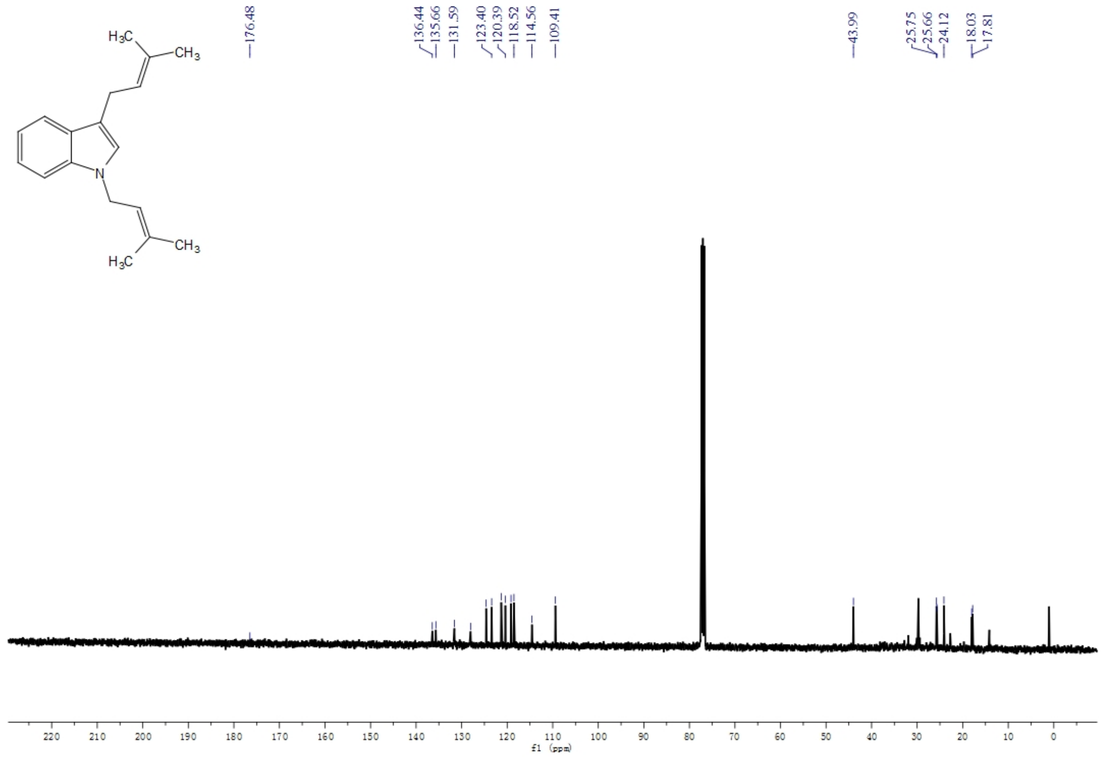


$^{13}$C-NMR spectrum of Compound **4**.

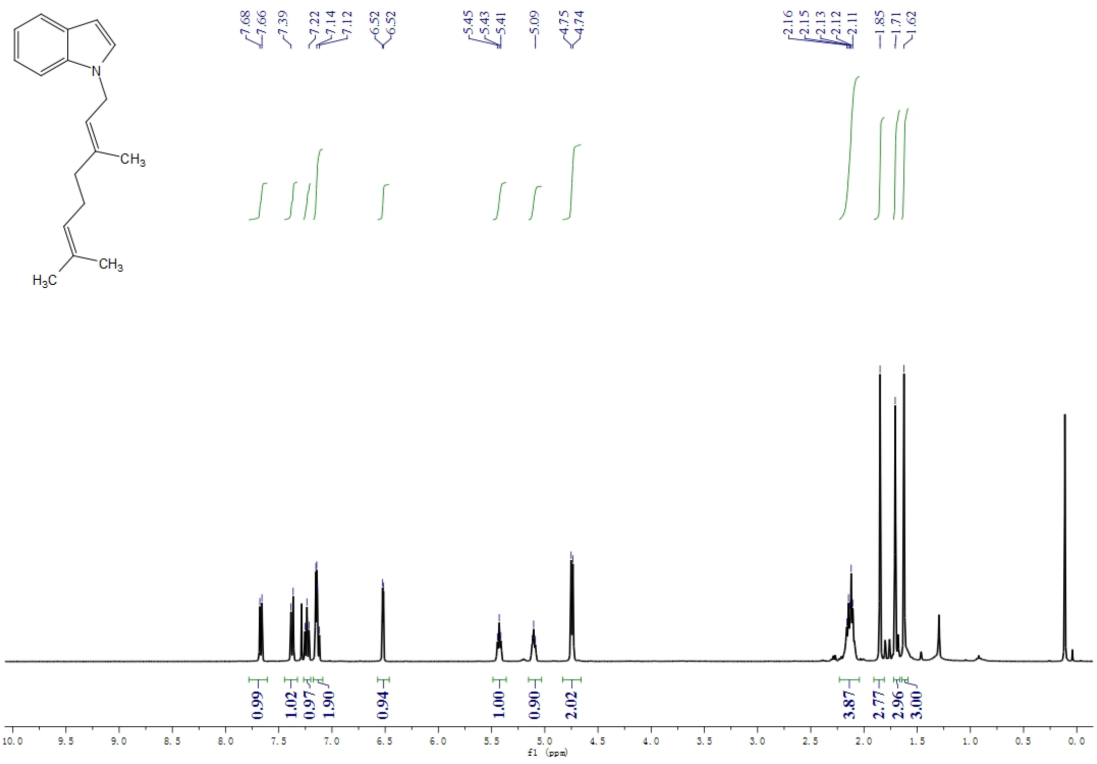

$^{1}$H-NMR spectrum of Compound **11**.

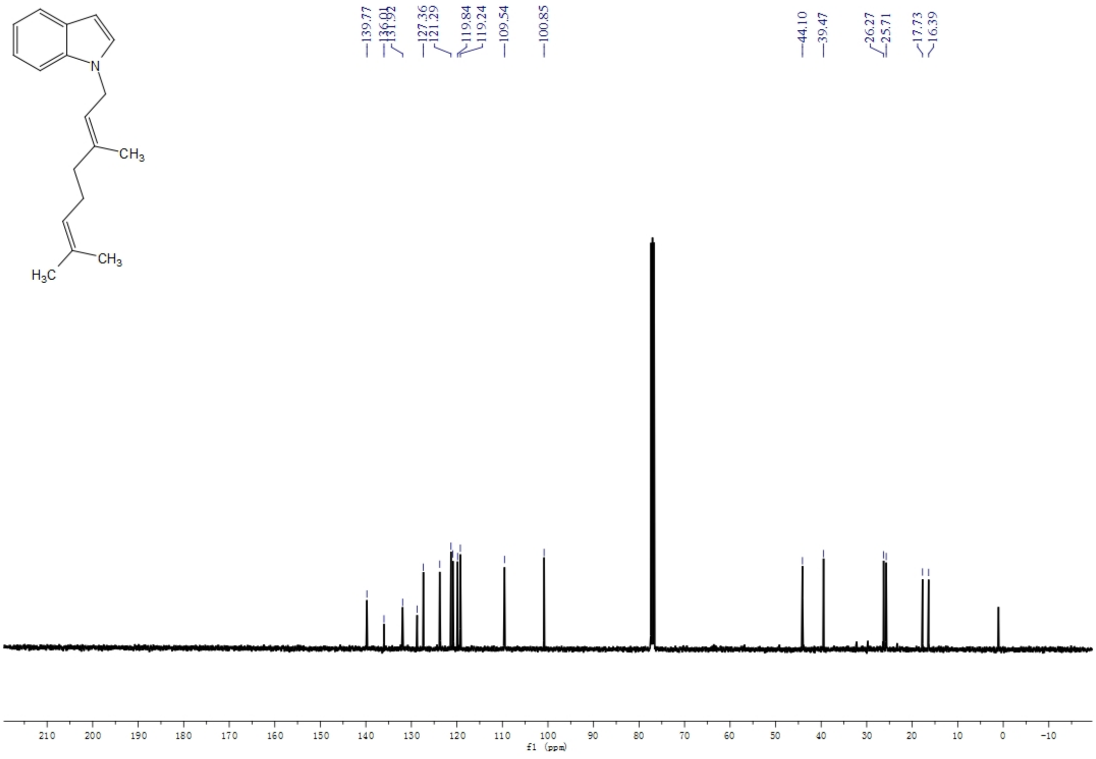

$^{13}$C-NMR spectrum of Compound **11**.

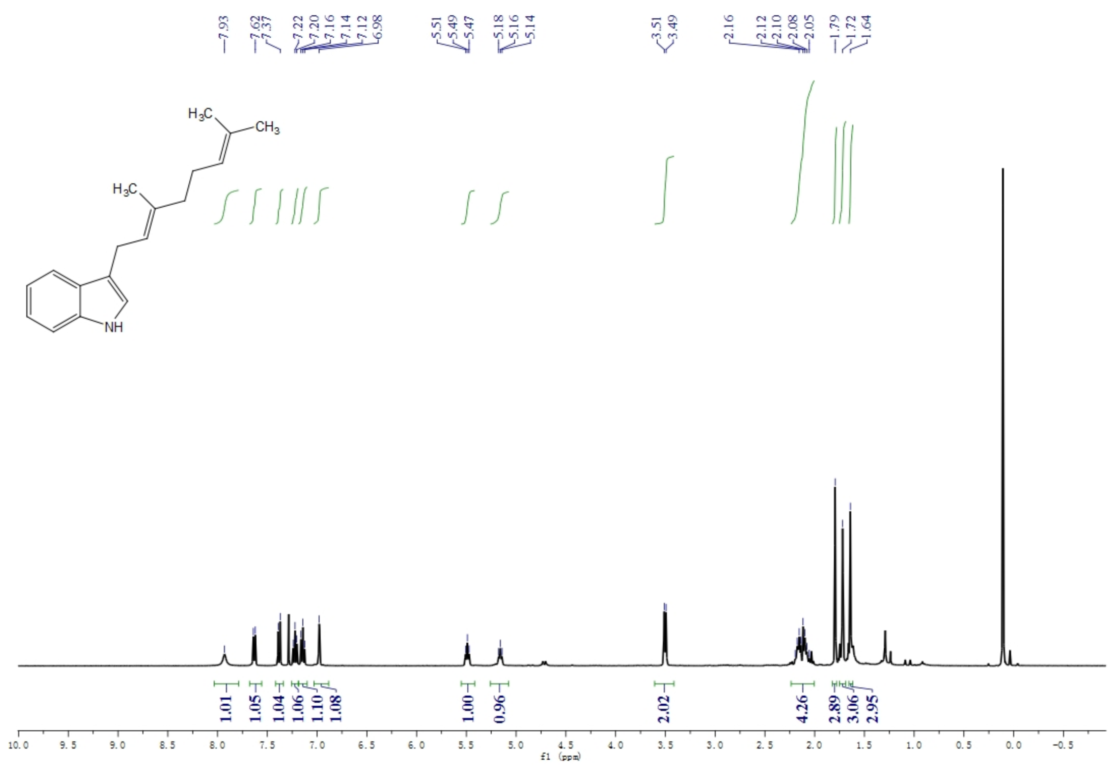


$^{1}$H-NMR spectrum of Compound **14**.

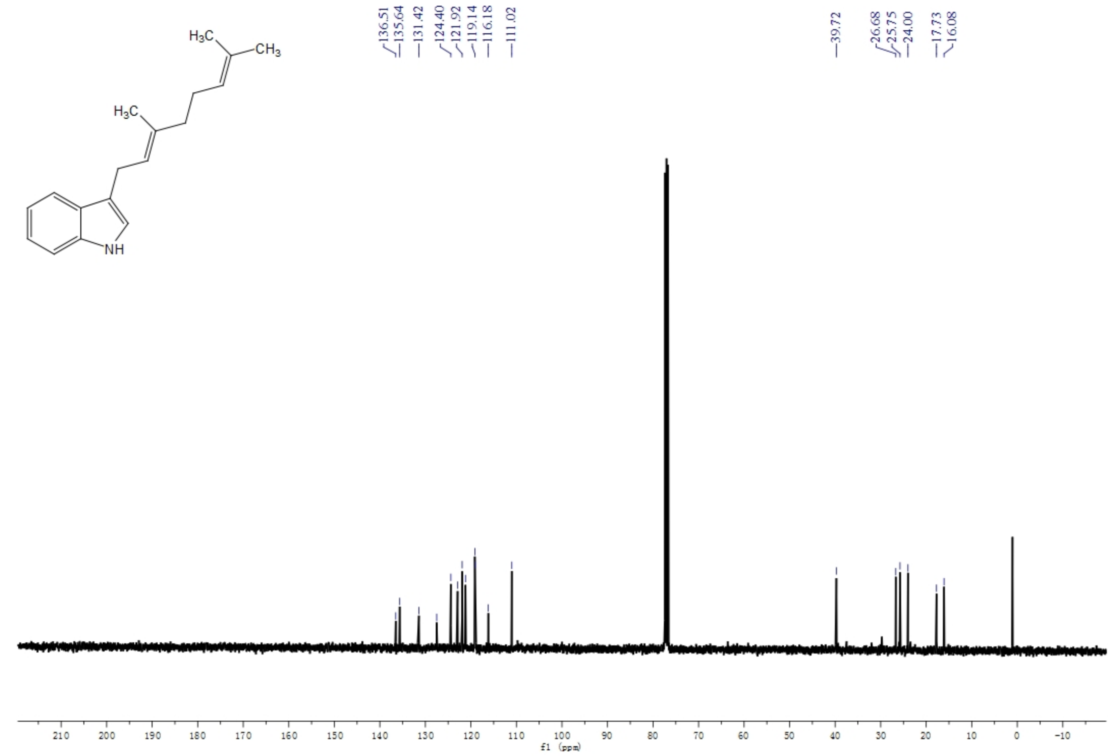


$^{13}$C-NMR spectrum of Compound **14**.

## Supplementary References


1. Ozaki, T., Mishima, S., Nishiyama, M. & Kuzuyama, T. NovQ is a prenyltransferase capable of catalyzing the addition of a dimethylallyl group to both phenylpropanoids and flavonoids. *J. Antibiot. (Tokyo).* **62**, 385–392 (2009).
2. Yu, X., Xie, X. & Li, S. M. Substrate promiscuity of secondary metabolite enzymes: Prenylation of hydroxynaphthalenes by fungal indole prenyltransferases. *Appl. Microbiol. Biotechnol.* **92**, 737–748 (2011).
3. Kumano, T., Richard, S. B., Noel, J. P., Nishiyama, M. & Kuzuyama, T. Chemoenzymatic syntheses of prenylated aromatic small molecules using Streptomyces prenyltransferases with relaxed substrate specificities. *Bioorganic Med. Chem.* **16**, 8117–8126 (2008).
4. Das, B., Veeranjaneyulu, B., Krishnaiah, M. & Balasubramanyam, P. Benzylation and allylation of naphthols using amberlyst-15. *Synth. Commun.* **39**, 1929–1935 (2009).
