# Supplementary material for: Discovery of the cryptic function of terpene cyclases as aromatic prenyltransferases
Source: Nat Commun. 2020 Aug 7;11:3958. doi: 10.1038/s41467-020-17642-2 (PMC7414894; doi:10.1038/s41467-020-17642-2)
Supplement: Supplementary file 2 — Descriptions of Additional Supplementary Files [file 41467_2020_17642_MOESM2_ESM.docx]

**Descriptions of Additional Supplementary Files**

**Supplementary Dataset 1**

**Description:** Sequences of terpene synthases used in this work.

**Supplementary Dataset 2**

**Description:** Data collection and refinement statistics.
